# Rescue of cardiomyopathy through U7snRNA-mediated exon skipping in *Mybpc3*-targeted knock-in mice

**DOI:** 10.1002/emmm.201202168

**Published:** 2013-05-29

**Authors:** Christina Gedicke-Hornung, Verena Behrens-Gawlik, Silke Reischmann, Birgit Geertz, Doreen Stimpel, Florian Weinberger, Saskia Schlossarek, Guillaume Précigout, Ingke Braren, Thomas Eschenhagen, Giulia Mearini, Stéphanie Lorain, Thomas Voit, Patrick A Dreyfus, Luis Garcia, Lucie Carrier

**Affiliations:** 1 Department of Experimental Pharmacology and Toxicology, Cardiovascular Research Center, University Medical Center Hamburg-EppendorfHamburg, Germany; 2 DZHK (German Centre for Cardiovascular Research)partner site Hamburg/Kiel/Lübeck, Germany; 3 Inserm U974, CNRS UMR7215, Institut de MyologieParis, France; 4 Pierre and Marie Curie University ParisParis, France; 5 Hamburg Zentrum für Experimentelle Therapieforschung (HEXT) Vector Core Unit, Department of Experimental Pharmacology and Toxicology, University Medical Center Hamburg-EppendorfHamburg, Germany

**Keywords:** alternative splicing, antisense oligoribonucleotide, cardiac myosin-binding protein-C, exon skipping, hypertrophic cardiomyopathy

## Abstract

Exon skipping mediated by antisense oligoribonucleotides (AON) is a promising therapeutic approach for genetic disorders, but has not yet been evaluated for cardiac diseases. We investigated the feasibility and efficacy of viral-mediated AON transfer in a *Mybpc3*-targeted knock-in (KI) mouse model of hypertrophic cardiomyopathy (HCM). KI mice carry a homozygous G>A transition in exon 6, which results in three different aberrant mRNAs. We identified an alternative variant (Var-4) deleted of exons 5–6 in wild-type and KI mice. To enhance its expression and suppress aberrant mRNAs we designed AON-5 and AON-6 that mask splicing enhancer motifs in exons 5 and 6. AONs were inserted into modified U7 small nuclear RNA and packaged in adeno-associated virus (AAV-U7-AON-5+6). Transduction of cardiac myocytes or systemic administration of AAV-U7-AON-5+6 increased Var-4 mRNA/protein levels and reduced aberrant mRNAs. Injection of newborn KI mice abolished cardiac dysfunction and prevented left ventricular hypertrophy. Although the therapeutic effect was transient and therefore requires optimization to be maintained over an extended period, this proof-of-concept study paves the way towards a causal therapy of HCM.

## INTRODUCTION

RNA-based therapeutics and splice-switching approaches have been developed over the last decade to treat cancer, asthma, rheumatoid arthritis or neuromuscular diseases such as Duchenne muscular dystrophy (DMD), spinal muscular atrophy and myotonic dystrophy type 1 (for reviews, see Aartsma-Rus et al, 2007; Aartsma-Rus and van Ommen, 2007; Le Roy et al, 2009; Lu et al, 2011). Several studies have shown successful restoration of the *DMD* reading frame by exon skipping using 2′-O-methyl phosphorothioate-(2OMePS)-antisense oligoribonucleotides (AONs) in mouse (*mdx*; Bremmer-Bout et al, 2004; Lu et al, 2003) and dog (golden retriever muscular dystrophy, GRMD; McClorey et al, 2006) disease models as well as in patient-derived muscle cell cultures (Aartsma-Rus et al, 2007; Madden et al, 2009; van Deutekom et al, [Bibr b54],[Bibr b55]), and recently by systemic administration in DMD patients (Goemans et al, 2011). However, although the exon skipping strategy using back-bone modified AONs has been extensively studied and is already validated in clinical trials, it shows limitations, such as the need for short-term repeated injections and lack of efficient exon skipping in the heart. This limitation may be circumvented by using viral-mediated gene transfer such as adeno-associated viral (AAV) vectors. Tail-vein administration of AAV encoding a modified U1 or U7 small nuclear RNA (snRNA) carrying antisense sequences that induce skipping of exon 23 restored and maintained dystrophin expression in cardiac and skeletal muscle of *mdx* mice until 74 weeks (Denti et al, 2008; Goyenvalle et al, [Bibr b22],[Bibr b23]). Similarly, percutaneous transendocardial delivery of AAV encoding U7snRNA carrying AON sequences directed against exons 6 plus 8 reduced fibrosis and improved cardiac function in the GRMD model after 13 months (Barbash et al, 2012; Bish et al, 2012). This promising strategy has not yet been evaluated for cardiac genetic diseases. The aim of the present study was therefore to investigate this approach in a mouse model of hypertrophic cardiomyopathy (HCM).

With a prevalence of 1:500 (Maron et al, 1995) HCM is the most frequent cardiac inherited disease and the leading cause of sudden cardiac death (SCD) under 35, particularly in young athletes (Maron et al, 1996). It is mainly characterized by left ventricular hypertrophy (LVH), diastolic dysfunction and increased interstitial fibrosis. It is also associated with a significant risk of heart failure and stroke in elderly (Ehlermann et al, 2008). The clinical outcome of HCM is highly variable and ranges from an asymptomatic benign course to heart failure, atrial fibrillation and SCD caused by arrhythmias (for reviews, see Elliott et al, 2008; Gersh et al, 2011). HCM is frequently caused by mutations in the *MYBPC3* gene encoding cardiac myosin-binding protein C (cMyBP-C; Olivotto et al, 2008; Richard et al, 2003; Van Driest et al, 2004), which is exclusively expressed in the heart (Fougerousse et al, 1998; Gautel et al, 1998). cMyBP-C is located in doublets in the C-zone of the A-band of the sarcomere, where it plays a major role (for reviews, see Barefield & Sadayappan 2010; Schlossarek et al, 2011). In particular, cMyBP-C tethers myosin-S2 to the thick filament and thereby limits myosin interaction with actin during diastole (Colson et al, 2007; Pohlmann et al, 2007). Furthermore, phosphorylation of cMyBP-C improves force of contraction by releasing the tether on the myosin lever arm (Sadayappan et al, 2005). About 61% of *MYBPC3* mutations are frameshift or nonsense mutations leading to truncated proteins. Findings in humans support the view that cMyBP-C haploinsufficiency is the major molecular mechanism of HCM (Marston et al, [Bibr b36],[Bibr b37]; Moolman et al, 2000; Rottbauer et al, 1997; van Dijk et al, [Bibr b56],[Bibr b57]). Findings in mice bearing a *Mybpc3* point mutation suggest that haploinsufficiency results from regulation by the nonsense-mediated mRNA decay (NMD), the ubiquitin–proteasome system (UPS) or both (Vignier et al, 2009). Recent data suggest that age or adrenergic stress leads to UPS impairment and potential accumulation of truncated proteins that could act as poison peptides (Schlossarek et al, 2012a,2012b).

The current clinical management of HCM is focused on relieving symptoms by pharmacological and/or surgical treatments, but does not address the cause of the disease. Here, we developed a vector-based exon skipping strategy to produce an in-frame modified mRNA and protein that was detected as a hitherto unknown variant in wild-type mice. The present study provides the first proof-of-principle evidence that AAV-U7-AONs remove a mutation in neonatal mouse cardiac myocytes (NMCMs) and *in vivo* in the heart of a HCM mouse model (*Mybpc3*-targeted knock-in (KI) mice). U7snRNA-mediated exon skipping increased the amount of the internally deleted but functional cMyBP-C variant, and, importantly, restored cardiac function and prevented LVH in newborn KI mice.

## RESULTS

### Evidence for a naturally spliced *Mybpc3* mRNA variant in knock-in and wild-type mice

**Figure 1:**
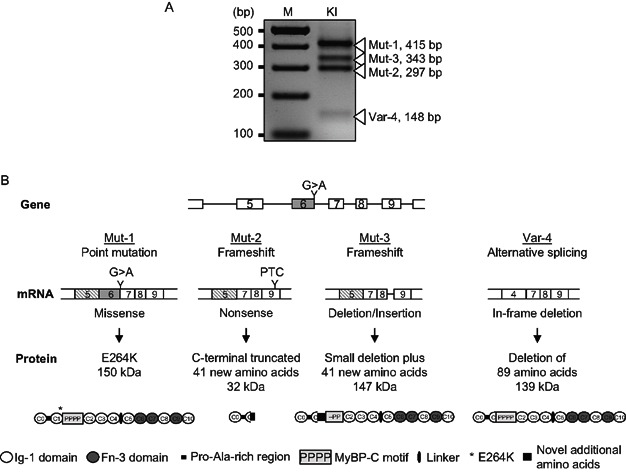
Molecular impact of the mutation in *Mybpc3*-targeted KI mice Representative RT-PCR from RNA of neonatal knock-in (KI) cardiac myocytes using primers located in exons 4 and 9 of *Mybpc3*. The different PCR products correspond to different mRNAs.Consequences of the G>A transition on the structure of *Mybpc3* mRNAs and cMyBP-C proteins. Three different mutant *Mybpc3* mRNAs (Mut-1 to Mut-3) and one spliced isoform (Var-4) were detected. Mut-1 *Mybpc3* mRNA (missense) contains the G>A transition and is expected to produce a full-length E264K mutant cMyBP-C protein. Mut-2 (nonsense) and Mut-3 (deletion/insertion) *Mybpc3* mRNAs are deleted of exon 6 and result in a frameshift. Whereas Mut-2 mRNA exhibits a PTC in exon 9 leading to a 32-kDa truncated protein, Mut-3 mRNA retains a part of intron 8 that restores the reading frame and produces a 147-kDa protein (Mut-3). Var-4 mRNA (alternative splicing) bears an in-frame deletion of exons 5 and 6, which is expected to produce a shortened 139-kDa protein. M, 100-bp molecular weight marker.

*Mybpc3*-targeted KI mice carry a G>A transition on the last nucleotide of exon 6 (Vignier et al, 2009), which is associated with a severe phenotype and bad prognosis in humans (Richard et al, 2003). This mutation was found in ∼13% of all HCM patients and in 30% of all *MYBPC3* mutation carriers in a large HCM cohort in Italy (Olivotto et al, 2008). Analysis of 10-week-old KI mice revealed higher myofilament Ca^2+^ sensitivity, diastolic and systolic dysfunction and LVH (Fraysse et al, 2012). Previous analysis of KI ventricular tissue indicated normal level of pre-mRNA, but markedly reduced levels of *Mybpc3* mutant mRNAs and proteins (Vignier et al, 2009). RT-PCR analysis of KI NMCMs with *Mybpc3*-specific primers revealed 4 different fragments (Fig 1). Three bands were previously described and correspond to mutants 1-3 (Vignier et al, 2009). Mutant 1 (Mut-1, missense mRNA) contains the G>A transition and is expected to produce a full-length 150-kDa E264K mutant protein. Mutant 2 (Mut-2, nonsense mRNA) is deleted of exon 6 and expected to result in a premature termination codon (PTC) in exon 9, resulting in a 32-kDa mutant protein. Mutant 3 (Mut-3) is deleted of exon 6, but retains part of intron 8 that restores the reading frame and produces a 147-kDa mutant protein. The newly detected band of 148 bp (variant-4; Var-4) corresponds to a mRNA that is in-frame deleted of exons 5 plus 6 and expected to result in a 139-kDa protein (Fig 1).

**Figure 2:**
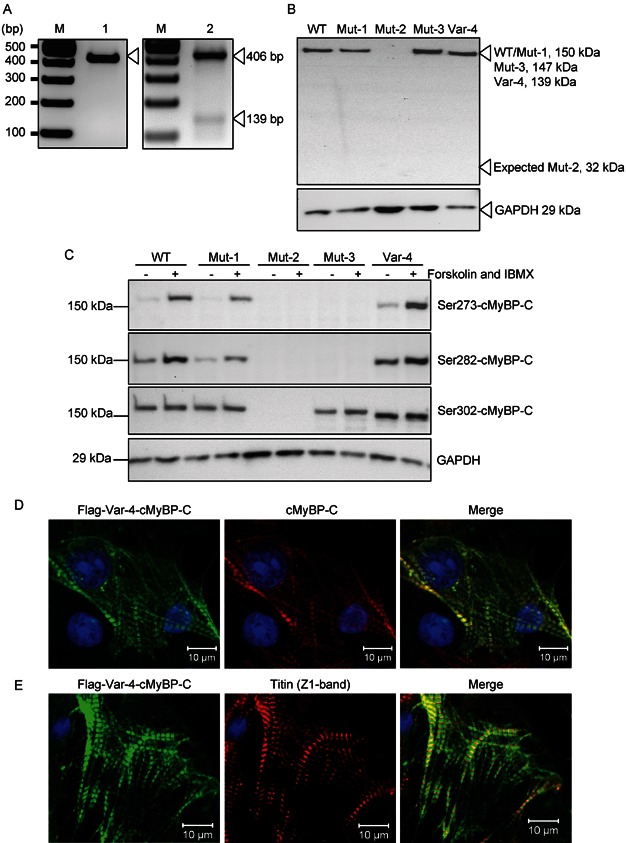
Expression and stability of the alternative spliced Var-4 *Mybpc3* isoform **A.** Representative RT-PCR from RNA extracted from neonatal wild-type (WT) cardiac myocytes after the first (1) and second (2) round of PCR using primers complementary to exons 4 and 7 of *Mybpc3*. The PCR product of the first round was used as a template for the second round. The expected fragment sizes are indicated by arrowheads.**B,C.** HEK293 cells were transiently transfected with plasmids encoding FLAG-tagged WT and mutant proteins (Mut-1 to Mut-3, Var-4) for 48 h in the absence (−) or presence (+) of forskolin (10 µM) and 3-Isobutyl-1-methylxanthine (IBMX, 250 µM). Western blots were either stained with an anti-FLAG antibody (B) or antibodies directed against different phosphorylation sites of cMyBP-C (C). GAPDH staining is shown in both panels. The expected molecular weights are indicated.**D,E.** Immunofluorescence analysis of neonatal KI cardiac myocytes transduced with an adeno-associated virus serotype 6 encoding FLAG-tagged Var-4 protein at a MOI of 5,000 for 48 h. Cells were double-stained with antibodies directed against the FLAG epitope and cMyBP-C (D) or the FLAG epitope and titin (Z1-domain, E). Nuclei were stained with TO-PRO-3 (in blue in the merged image). The right panels represent higher magnifications of the merged images (white rectangle). M, 100-bp molecular weight marker.

To investigate whether Var-4 is a naturally occurring alternative mRNA isoform, RNA from wild-type (WT) NMCMs was used for two rounds of PCR amplification with primers complementary to exons 4 and 7 (Fig 2). After the first round of PCR the expected 406-bp fragment was obtained in WT. The second round of PCR revealed an additional 139-bp fragment, which corresponded to the fusion of exon 4 with exon 7 (Supporting Information Fig S1), suggesting that Var-4 is an alternative spliced isoform present at low level in WT mice. Var-4 mRNA was detected in NMCMs isolated from WT mice (C57BL/6J) and in ventricular tissue of WT and KI mice (either C57BL/6J or Black swiss) during the entire development (Supporting Information Fig S2). To further evaluate the stability and phosphorylation of Var-4 protein, HEK293 cells were transiently transfected with plasmids encoding FLAG-tagged WT, Mut-1, Mut-2, Mut-3 and Var-4 cMyBP-Cs. Var-4 protein level did not differ from WT, Mut-1 and Mut-3, suggesting that it is very stable. On the other hand, Mut-2, expected at 32 kDa, was not detected, supporting previous findings of its rapid degradation by the UPS (Fig 2; Sarikas et al, 2005). We then investigated whether Var-4 is phosphorylated by PKA. Transfected HEK293 cells were cultured in the presence or absence of forskolin and 3-isobutyl-1-methylxanthine, and cMyBP-C phosphorylation was evaluated using antibodies directed against Ser-273, Ser-282 and Ser-302 residues. The basal level of phosphorylated cMyBP-C did not differ between Var-4, Mut-1 and WT (Fig 2). Phosphorylation of Mut-3 was only detected at Ser-302, as expected from the frameshift (Fig 2). Activation of PKA increased the phosphorylated cMyBP-C level at Ser-273, Ser-282, and to a lower extent at Ser-302 in Var-4, Mut-1 and WT, and only at Ser-302 in Mut-3 (Fig 2). Finally, we investigated whether Var-4 protein is incorporated into the sarcomere. KI NMCMs were transduced with an AAV serotype 6 (AAV6) encoding the FLAG-tagged Var-4 protein. Analysis was performed by confocal microscopy using antibodies directed against cMyBP-C, titin and the FLAG epitope (Fig 2). FLAG-Var-4 protein exhibited a striated pattern, was co-localized with cMyBP-C, but not with titin. This suggests that Var-4 protein is well incorporated into the A-band of the sarcomere.

Together, these data suggest that Var-4 protein is likely functional and not toxic. We therefore hypothesized that enhancing its expression would increase the level of cMyBP-C in the sarcomere and therefore ameliorate cardiac function.

### Antisense oligoribonucleotides efficiently induce exon skipping in cardiac myocytes

**Figure 3:**
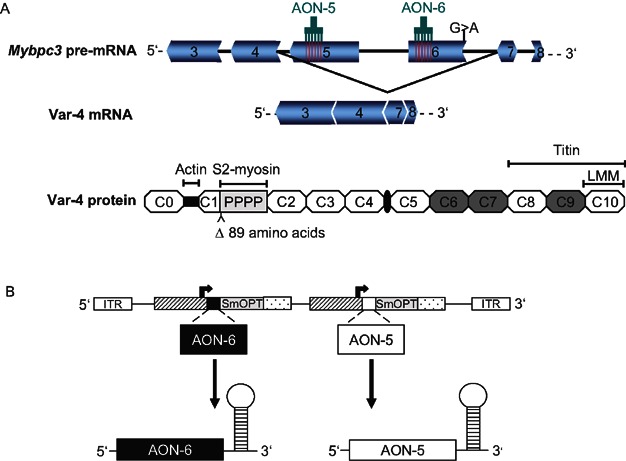
Exon skipping strategy and construct encoding U7 modified snRNA carrying AON-5+6 Principle of the exon skipping strategy to remove exons 5 and 6 in *Mybpc3* mRNA using AONs. The AONs are complementary to ESE motifs (red lines) in exon 5 (AON-5) and/or exon 6 (AON-6) of *Mybpc3* (upper drawing). They are expected to induce skipping of the corresponding exons (middle panel). The resultant protein lacks (Δ) 89 amino acids of the C1 domain, but still contains the binding domains to actin, titin, S2-myosin and light-meromyosin (LMM), as well as an intact MyBPC-motif containing the 4 phosphorylation sites (PPPP; lower panel). LMM, light meromyosin.Upper panel: Structure of the adeno-associated viral vector modified snRNA carrying AON-6 or AON-5 under the control of two independent U7 promoters. The two U7 cassettes were cloned in tandem between two inverted terminal repeats (ITRs). One cassette encodes the modified U7 snRNA gene along with its natural promoter (black arrow, hatched box), the antisense sequence (black or open box), the canonical Sm sequence (SmOPT, gray box) and 3′ elements (dotted box). Lower panel: Modified snRNAs carrying AON-6 and AON-5 sequences.

To enhance the expression of Var-4, we designed AONs that mask exonic splicing enhancer (ESE) motifs in exon 5 (AON-5) and in exon 6 (AON-6) of *Mybpc3*, and are therefore expected to induce an in-frame exon skipping (Fig 3). Since the mutation also results in the skipping of exon 6 (Mut-2 and Mut-3; Fig 1), we assumed that AON-5 or AON-5 plus AON-6 (AON-5+6), but not AON-6 alone would induce skipping of both exons in KI NMCMs. The specificity of the designed AONs was evaluated in WT and KI NMCMs using AON-5 or AON-5+6. Each AON was full-length modified by 2OMePS groups to confer nuclease resistance. Analysis was performed by RT-PCR 8 days after transfection. Whereas treatment of WT NMCMs with AON-5 resulted in the skipping of exon 5 only, treated KI NMCMs presented the skipping of both exons. Treatment with AON-5+6 together resulted in skipping of both exons in WT and KI NMCMs (Supporting Information Fig S3A and S3B). In KI NMCMs exon skipping was accompanied by reduced amounts of mutant mRNAs. Skipping of exons 5 and 6 resulted in the accumulation of Var-4 protein by Western blot (Supporting Information Fig S3C). These results indicate that the exon skipping strategy works in NMCMs and results in specific skipping of the targeted exons.

**Figure 4:**
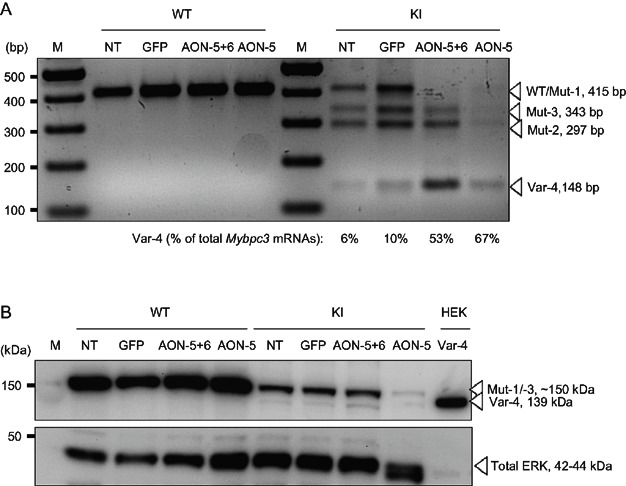
Effect of AAV6-U7-AONs on *Mybpc3* mRNAs and cMyBP-C proteins in cardiac myocytes Cardiac myocytes isolated from neonatal wild-type (WT) or knock-in (KI) mice were either not-transduced (NT) or transduced with adeno-associated virus serotype 6 (AAV6) encoding GFP (GFP), U7-AON-5 (AON-5), or U7-AON-5+6 (AON-5+6) at a MOI of 10,000. RT-PCR using primers located in exons 4 and 9 of *Mybpc3*. The level of mutant *Mybpc3* KI mRNAs was quantified using the Gene Tool Software (Syngene, Cambridge) and the Var-4 mRNA level was expressed as percentage of total *Mybpc3* mRNAs and indicated in the figure.Western blot stained with antibodies directed against the N-terminus of cMyBP-C or total ERK. M, 100-bp molecular weight marker; ERK, extracellular signal-related kinase; HEK-Var-4, HEK 293 cells transfected with a plasmid encoding Var-4.

For further experiments, we produced AAV encoding modified U7snRNA (Goyenvalle et al, 2004) carrying either AON-5 alone or AON-5+6 sequences (Fig 3). U7 is a non-spliceosomal snRNA, which is normally involved in histone pre-mRNA 3′-end processing (Galli et al, 1983). By a small change in the sequence for the binding site of Sm/Lsm proteins (SmOPT; Stefanovic et al, 1995), it can be redirected to the spliceosome and used as a shuttle for antisense sequences. Here, the sequence was embedded into a snRNP particle and thus protected from degradation. WT and KI NMCMs were transduced with AAV6 encoding GFP or U7-AON-5+6 at a MOI of 10,000 or were not-transduced (NT). The proportion of Var-4 mRNA was 5–6-fold higher in KI NMCMs transduced with U7-AON-5 or with U7-AON-5+6 than in NT or GFP-transduced KI NMCMs (Fig 4). This was accompanied by marked reduction or disappearance of Mut-1 mRNA. On the other hand, whereas U7-AON-5 or U7-AON-5+6 did not induce exon skipping in WT NMCMs 48 h after transduction (Fig 4), it did so 72 h after transduction, but the extent was much less than in KI (Supporting Information Fig S4). Nonsense *Mybpc3* mRNA deleted of either exon 5 or exon 6 was stabilized by the translational inhibitor emetine, indicating that they are normally degraded by the NMD in WT cells (Supporting Information Fig S4). We next investigated the efficiency of exon skipping at the protein level by Western blot (Fig 4). As expected from the mRNA analysis, no Var-4 protein was detected in WT NMCM transduced with U7-AON-5 or U7-AON-5+6 48 h after transduction. On the other hand, the proportion of Var-4 protein was higher in KI NMCM transduced with U7-AON-5 or U7-AON-5+6 than in NT KI NMCMs or those transduced with GFP (Fig 4).

### AAV9-U7-AON-5 + 6 induces efficient exon skipping in the heart of 4-week-old KI mice

**Figure 5:**
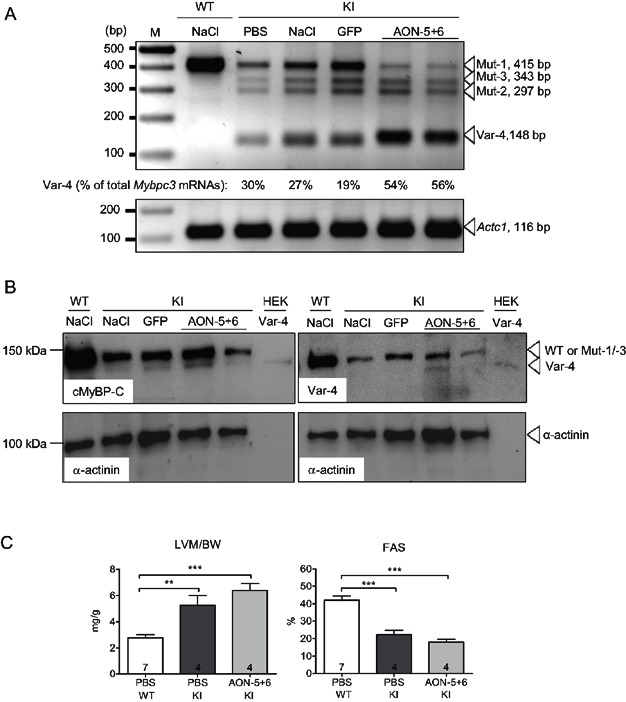
Impact of AAV9-U7-AONs on molecular and functional phenotype in 4-week-old mice KI mice received NaCl, PBS, adeno-associated virus serotype 9 (AAV9) encoding GFP (GFP, 7.6 × 10^10^ vg) or encoding U7-AON-5+6 (9.4 × 10^11^ vg) by systemic administration into the tail vein. Analyses of ventricular tissue were performed 4 weeks post-injection. RT-PCR analysis using primers located in exons 4 and 9 of *Mybpc3* and α-cardiac muscle actin (*Actc1*). The level of mutant *Mybpc3* mRNAs was quantified using the Gene Tool Software (Syngene, Cambridge) and the level of Var-4 mRNA was expressed as percentage of total *Mybpc3* mRNAs, and indicated in the figure.Western blot stained with antibodies directed against the N-terminus of cMyBP-C (cMyBP-C), the amino acids produced by the fusion of exon 4 with exon 7 (Var-4) or against α-actinin. The Var-4-cMyBP-C antibody also detects WT, Mut-1 and/or Mut-3 cMyBP-C proteins. As positive controls, protein extracts from either ventricular tissue of a wild-type mouse injected with PBS (WT-NaCl) or from HEK293 cells transfected with a plasmid encoding Var-4 were used (HEK-Var-4). The expected fragments are indicated by arrowheads.Echocardiographic analyses were performed 4 weeks after administration of AAV9 or PBS in KI and/or WT mice. Fractional area shortening (FAS), left ventricular mass-to-body-weight (LVM/BW) ratio are shown. Data are expressed as mean ± SEM. **p < 0.01 and **p < 0.001 *versus* PBS-treated WT, one-way ANOVA and Bonferroni post-hoc test. Number of animals is indicated in the bars. M, 100-bp molecular weight marker.

To evaluate the efficiency of exon skipping in the mouse heart *in vivo*, we used AAV serotype 9 (AAV9), which has been shown to have a high cardiotropism in mice (Inagaki et al, 2006). Phosphate buffered saline (PBS), sodium chloride (NaCl), or AAV9 encoding GFP or U7-AON-5+6 were administered in 4-week-old KI mice by tail-vein injection. As a further control, one WT mouse received NaCl. The expression of GFP was evaluated by RT-PCR in different tissues (heart, lung, liver and kidney) 3–5 weeks after injection. GFP mRNA level was markedly higher in the heart than liver, lung and kidney (Supporting Information Fig S5), confirming the cardiotropism of AAV9. Systemic administration of U7-AON-5+6 (9.4 × 10^11^ viral genome (vg)/mouse, which corresponds to a mean dose of 4.8 × 10^13^ vg/kg of body weight [BW]) markedly induced the skipping of exons 5 plus 6 and resulted in a higher level of Var-4 mRNA than in control mice 4 wks after (∼55% of total *vs*. a mean of 25% in controls; Fig 5). This was accompanied by reduced amounts of Mut-1 mRNA. To detect Var-4 protein we used two different antibodies, one directed against the N-terminal domain of cMyBP-C (cMyBP-C antibody) and the second one directed against the amino acids produced by the fusion of exon 4 with exon 7 (Var-4 antibody). Both revealed that U7-AON-5+6 increased the Var-4 protein level when compared to PBS or GFP-injected KI mice (Fig 5).

Cardiac function was evaluated by echocardiography in WT mice that had received PBS and in KI mice that had received PBS or AAV9 encoding U7-AON-5+6 4 weeks before. Compared to WT animals, KI mice treated with PBS exhibited lower fractional area shortening (FAS) and higher left ventricular mass-to-BW ratio (LVM/BW; Fig 5). The cardiac phenotype was not improved after U7-AON5 + 6 administration. This suggests that (i) LVH and cardiac dysfunction cannot be rescued in KI mice at this stage or (ii) that the dose of virus was too low.

### AAV9-U7-AON-5 + 6-mediated exon skipping restores cardiac function and prevents left ventricular hypertrophy in newborn KI mice

**Figure 6:**
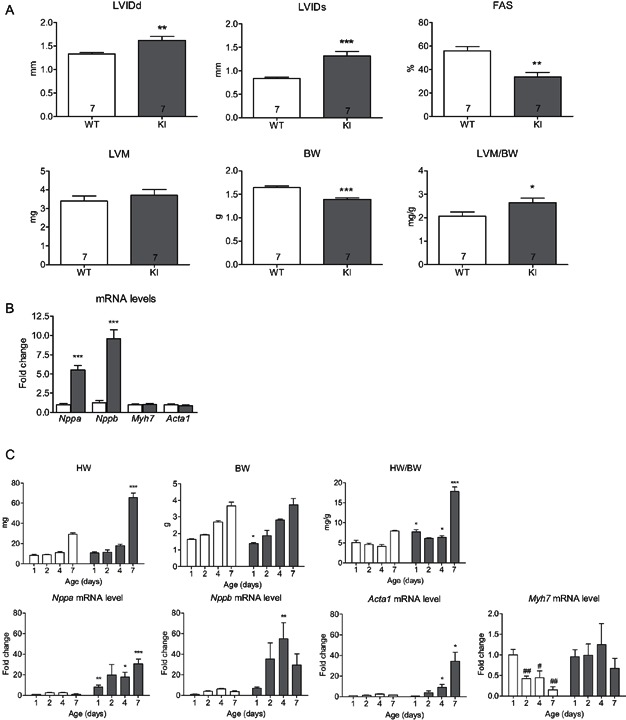
Evaluation of the cardiac and molecular phenotype in neonatal mice Echocardiographic analyses were performed in 1-d-old WT and KI mice. Data are expressed as mean ± SEM. *p < 0.05, **p < 0.01 and **p < 0.001 *versus* WT, Student's *t*-test (*n* = 7).RT-qPCR of hypertrophic markers in ventricular tissue of 1-day-old KI and WT mice. Data are expressed as mean ± SEM, ***p < 0.001 *versus* WT, Student's *t*-test (*n* = 7).Evaluation of the cardiac phenotype and RT-qPCR of hypertrophic markers in ventricular tissue of 1-7-day-old KI and WT neonatal mice. Data are expressed as mean ± SEM. *p < 0.05, **p < 0.01, and ***p < 0.001 *versus* WT, same age, and ^#^*p* < 0.05 and ^##^*p* < 0.01 *versus* 1-day-old WT, two-way ANOVA Bonferroni *post hoc* test (*n* = 4). Gray and white bars correspond to KI and WT mice, respectively. *Acta1*, α-skeletal actin; BW, body weight; FAS, fractional area shortening; HW, heart weight: LVIDd, left ventricular internal diameter in diastole; LVIDs, left ventricular internal diameter in systole; LVM, left ventricular mass; *Nppa*, atrial natriuretic peptide; *Nppb*, brain natriuetic peptide; *Myh7*, β-myosin-heavy chain.

To investigate these hypotheses further, we used newborn mice. Echocardiography was first performed in untreated 1-d-old WT and KI mice (Fig 6). KI mice exhibited higher left ventricular internal diameter (LVID) both in diastole and in systole, which resulted in lower FAS than in WT mice. LVM did not differ between the groups, but BW was lower in KI than in WT mice. Therefore the resulting higher LVM/BW was not the result of LVH in KI mice. These data indicate that 1-d-old KI mice exhibit only LV dilation and dysfunction without LVH. Analysis of the foetal gene program revealed higher mRNA levels for atrial natriuretic peptide (*Nppa*) and brain natriuretic peptide (*Nppb*) in 1-d-old KI than WT mice, whereas β-myosin heavy chain (*Myh7*) and α-skeletal actin (*Acta1*) mRNA levels did not differ among the groups (Fig 6). In order to determine when LVH started in KI mice, we evaluated KI and WT mice at postnatal days 1, 2, 4 and 7 (Fig 6). Heart weight (HW) and therefore HW-to-body weight ratio (HW/BW) started to be higher at day 4 and were both markedly higher at day 7 in KI than WT mice. This was paralleled by the accumulation of *Acta1* mRNA in KI at day 4. Interestingly, *Myh7* mRNA level remained high in KI whereas it decreased quickly after birth in WT mice.

**Figure 7:**
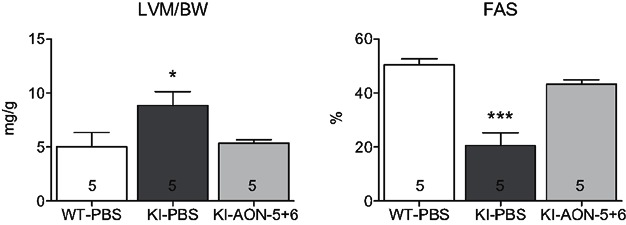
Impact of AAV9-U7-AON-5+6 on cardiac function in newborn mice 1-2-day-old KI mice received PBS or adeno-associated virus serotype 9 (AAV9) encoding U7-AON-5+6 (2 × 10^11^ vg) by systemic administration into the temporal vein. Echocardiographic analyses were performed 7 days post-injection. Data are expressed as mean ± SEM. *p < 0.05 and ***p < 0.001 *versus* PBS-treated WT, one-way ANOVA and Dunnett *post hoc* test. Number of animals is indicated in the bars. BW, body weight; FAS, fractional area shortening; LVM, left ventricular mass.

**Figure 8:**
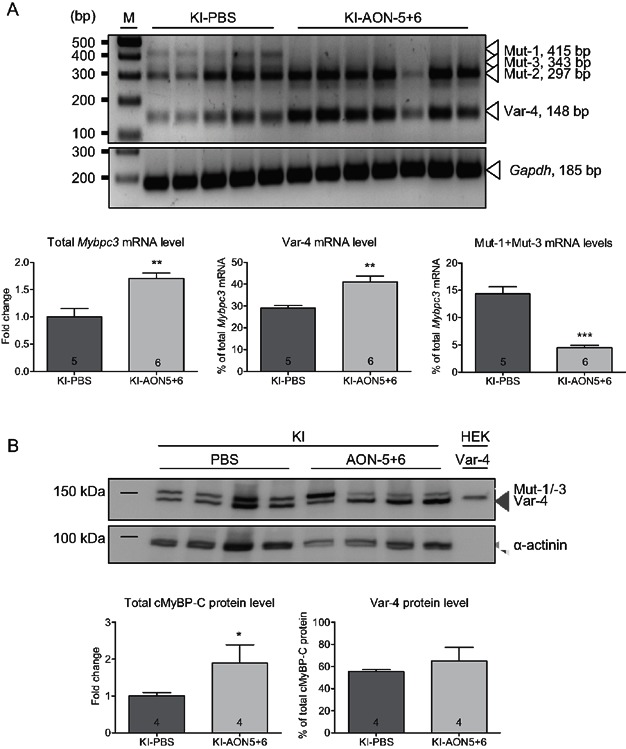
Evaluation of exon skipping efficacy 7 days after AAV9-U7-AON-5+6 administration in newborn mice 1-day-old KI mice received PBS or adeno-associated virus serotype 9 (AAV9) encoding U7-AON-5+6 (2 × 10^11^ vg) by systemic administration into the temporal vein for 7 days. RT-PCR using primers located in exons 4 and 9 of *Mybpc3* or in *Gapdh*. Determination of the mRNA levels was performed by densitometry (Gene Tool Sofware; Syngene, Cambridge). Total *Mybpc3* mRNA level was normalized to *Gapdh* mRNA level and related to KI-PBS. Var-4 and Mut-1 mRNA levels were expressed as percentage of total *Mybpc3* mRNA.Western blot stained with antibodies directed against the MyBP-C motif of cMyBP-C or α-actinin. Total cMyBP-C level was normalized to α-actinin and related to KI-PBS, and Var-4 protein level was expressed as a percentage of total cMyBP-C protein level. Var-4 positive control corresponds to protein extract from HEK293 cells transfected with Var-4 cDNA. Data are expressed as mean ± SEM. *p < 0.05, **p < 0.01 and ***p < 0.001 *versus* PBS-treated KI, Student's *t*-test. Number of animals is indicated in the bars. M, 100-bp molecular weight marker.

Then, we evaluated whether U7-AON-5+6 prevents LVH and rescues cardiac function in neonatal KI mice. AAV9 encoding U7-AON-5+6 was administered into the temporal vein (Dominguez et al, 2011) of 1-2-day-old KI mice and results were compared to KI and WT mice that received PBS. Each mouse received 2 × 10^11^ vg of AAV9 corresponding to a mean dose of 1.44 × 10^14^ vg/kg BW. This relative dose was threefold higher than the dose administered in 4-week-old mice. Evaluation of GFP expression by immunofluorescence in cardiac sections suggested high transduction efficiency 7 days after injection (Supporting Information Fig S5). Echocardiography was performed 7 days after U7-AON-5+6 administration (Fig 7). As expected, LVM/BW was higher and FAS lower in PBS-treated KI than in WT mice. Strikingly, both parameters were completely rescued in KI mice that received U7-AON-5+6, and therefore did not differ from WT parameters. To evaluate whether the functional improvement was associated with exon skipping, another series of 1-d-old KI mice was treated with U7-AON-5+6 or PBS for 7 days, and molecular analyses were performed in ventricular tissue (Fig 8). RT-PCR showed higher Var-4 and Mut-2 mRNA levels, lower levels of Mut-1 + Mut-3 mRNA, and higher levels of total *Mybpc3* mRNA in the U7-AON-5+6- than PBS-treated group (Fig 8). Western blot analysis with a cMyBP-C antibody directed against the MyBP-C motif performed on Urea protein fractions revealed two bands in both groups, corresponding to Mut-1/-3 and to Var-4, respectively. A shift was observed towards the Var-4 isoform and higher level of total cMyBP-C in most of the mice that received U7-AON-5+6 (Fig 8). Immunofluorescence analysis of mouse heart sections did not show major differences in the sarcomeric pattern between PBS- and U7-AON-5+6-treated mice (Supporting Information Fig S6).

To assess whether the higher level of Var-4 could be the cause of the improved cardiac phenotype, we evaluated the consequence of systemic delivery of AAV9 encoding FLAG-Var-4 in 1-d-old KI mice 7 days after administration (Supporting Information Fig S7). FLAG-Var-4 mRNA and protein were detected in the heart 7 days after AAV9 administration. Echocardiographic analysis revealed lower LVM/BW and higher FAS in Var-4- than PBS-treated KI mice. This was accompanied by the complete inactivation of the expression of the foetal genes, except for *Myh7*. This indicates that Var-4 functionally substitutes for WT cMyBP-C.

**Figure 9:**
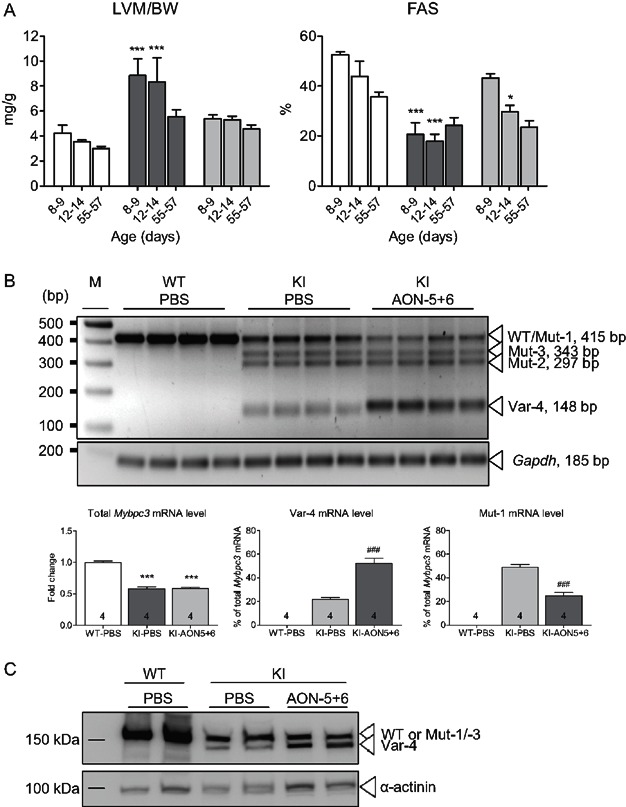
Long-term effect of AAV9-U7-AON-5+6 on the functional and molecular phenotype 1-2-day-old KI mice received PBS or adeno-associated virus serotype 9 (AAV9) encoding U7-AON-5+6 (2 × 10^11^ vg) by systemic administration into the temporal vein. Echocardiographic analyses were performed at different postnatal windows from 7 days to 55 days post-injection (*n* = 3–6).RT-PCR using primers located in exons 4 and 9 of *Mybpc3* or in *Gapdh*. Total *Mybpc3* mRNA level was normalized to *Gapdh* and related to WT. Determination of the mRNA levels was performed by densitometry (Gene Tool Software; Syngene, Cambridge) and Var-4 *Mybpc3* mRNA level was expressed as a percentage of total *Mybpc3* mRNA. Data are expressed as mean ± SEM. ***p < 0.001 *versus* PBS-treated WT, and ^###^*p* < 0.001 *versus* PBS-treated KI, one-way ANOVA and Bonferroni *post hoc* test. Number of animals is indicated in the bars.Western blot stained with antibodies directed against the MyBP-C motif of cMyBP-C or α-actinin. BW, body weight; FAS, fractional area shortening; LVM, left ventricular mass: M, 100-bp molecular weight marker.

We finally evaluated whether the rescue of the functional and molecular phenotype persisted over a longer period of time (Fig 9). In AAV9-treated mice, LVM/BW remained stable and lower than in PBS-treated KI mice over the observation period of 55 days after AAV9-U7-AON-5+6 injection, but FAS decreased (Fig 9). This pattern reflected that of WT mice at a slightly lower level, but clearly differed from PBS-treated KI mice, which started with low FAS that rather increased with time. On the other hand, the skipping of exons 5 plus 6 was persistent and resulted in a 2-fold higher level of Var-4 mRNA than in PBS-treated KI mice (Fig 9). This was accompanied by reduced amounts of Mut-1 mRNA. Var-4 protein was also stabilized 55 days after treatment (Fig 9). In order to understand why the rescue was not fully maintained over time, we analysed viral particle density in the heart (Supporting Information Fig S8). Virus genome was detected only in mice, which received AAV9 and the quantity was 4-fold lower 55 days than 7 days after administration. This may suggest the need for higher virus doses to maintain functional rescue.

## DISCUSSION

This is the first proof-of-principle study demonstrating that AONs induce exon skipping and the production of in-frame modified mRNA and protein in isolated cardiac myocytes and in the heart *in vivo* in a *Mybpc3*-targeted KI mouse model of HCM. The major findings of the present study are: (i) a new alternative spliced isoform of *Mybpc3* deleted of exons 5 and 6 (Var-4) was identified in both WT and KI mice; (ii) gene transfer of Var-4 in HEK293 cells and in NMCMs resulted in a stable protein, phosphorylated by PKA, and correctly incorporated into the sarcomere, (iii) Var-4 expression was increased using AONs directed against ESE motifs located in exons 5 and 6 of *Mybpc3* in cardiac myocytes; (iv) systemic administration of AAV9 encoding modified U7snRNA carrying AON-5+6 induced accumulation of Var-4 mRNA and protein in KI mice; v) importantly, systemic delivery of AAV9 encoding U7-AON-5+6 abolished systolic dysfunction and prevented development of LVH in neonatal KI mice. Although the therapeutic effect was transient, this study represents the first step towards molecular-based therapy of HCM.

Although there are some reports of exon skipping in the heart of animal models of DMD, this strategy was never evaluated in the context of HCM, and more generally in the context of cardiac genetic diseases. In our study, analyses were performed in homozygous KI mice, which bear a *Mybpc3* point mutation that is located in the consensus splice donor site sequence of exon 6. This mutation was found in ∼13% of HCM patients and represents therefore the most frequent HCM mutation, at least in Italy (Olivotto et al, 2008). Characterization of KI mice revealed three mutant mRNAs (Vignier et al, 2009). In the present study, we identified a new mRNA isoform lacking exons 5 and 6 in NMCMs and ventricular tissue of KI and WT mice of different genetic backgrounds (Figs 1 and 2, and Figs S1 and S2). This suggests the existence of a naturally spliced *Mybpc3* mRNA isoform, which was not described in the literature and that we name Var-4. Our data indicate stability, normal phosphorylation and incorporation of Var-4 protein into the sarcomere (Fig 2). Var-4 could thus be a “therapeutic molecule” that keeps its regulating properties during contraction and relaxation. We hypothesized therefore that strategies aiming at increasing its level could rescue the molecular and functional phenotype.

The only study using 2OMePS-AONs in NMCMs was described recently (Wang et al, 2010). The authors established a cardiac myocyte model to screen AONs for skipping of the mutated exon 23 in *mdx* mice, a model of DMD, and revealed low skipping efficiency (∼5%). Other studies investigated systemic delivery of 2OMePS-AONs in *mdx* mice and revealed a very low or rather non-existent exon skipping efficiency in the heart (Heemskerk et al, 2009; Lu et al, 2005). Direct intra-cardiac injection of naked morpholino AONs resulted in local exon skipping and dystrophin production, but at much lower levels than observed after intramuscular injection in skeletal muscles of *mdx* mice (Vitiello et al, 2008). This observation was likely due to the fact that skeletal muscle fibers of *DMD* mutation-carriers show damages and membrane tears and are thus more diffusible than heart cells, which are less affected. In the present study, 2OMePS-AONs were used to test the targeting specificity of the designed AON sequences. We show that transfection of cardiac myocytes with 2OMePS-AON-5 or -AON-5+6 both induced skipping of the corresponding exons from WT *Mybpc3* mRNA ( Fig S2). Interestingly, KI NMCMs treated either with AON-5 or AON-5+6 increased the production of Var-4 mRNA, and this was associated with reduced amounts of mutant 1–3 mRNAs. We suggest that the mutation on the consensus splice donor site of exon 6 destroys an ESE motif, which results in a weak splicing signal followed by the skipping of exon 6 together with the targeted exon 5.

Transduction of KI NMCMs with AAV6 encoding U7-AON-5 or U7-AON-5+6 markedly increased the amount of Var-4 mRNA (Fig 4). This completely prevented the formation of Mut-1 mRNA, whereas Mut-2 and Mut-3 mRNA remained. We suggest that besides the skipping of both exons, U7-AON-5+6 also leads to the skipping of individual exon, and thus skipping of only exon 6 would result in Mut-2 and/or Mut-3 mRNAs. This is further supported by the detection of mRNA deleted of exon 5, exon 6 or both exons 5 + 6 after administration of 2OMePS-U7-AON-5+6 or AAV6-U7-AON-5+6 in WT cells (Figs S3 and S4) and by the detection of higher levels of both Var-4 and Mut-2 mRNAs after AAV9-U7-AON-5+6 injection *in vivo* (Figs 8 and 9). Previous data obtained in myotubes of DMD patients also showed that AONs directed against two different exons result in the skipping of both exons as well as the skipping of the single exons (Aartsma-Rus et al, 2004). On the other hand, this finding may be explained by a potential competition of targeting exon 5 and/or exon 6, which results in the skipping of either both exons, exon 5 or exon 6 in some cells. The single skipping of exon 5 is expected to result in a frameshift and a PTC in exon 6. This mRNA was unstable and degraded by the NMD ( Fig S3). Interestingly, transduction of WT NMCM with AAV6-U7-AON-5+6 did not result in Var-4 mRNA or protein after 48 h and only in low levels after 72 h ( Fig S4). This suggests that double skipping is less effective in WT cells and that single exon-skipped mRNAs are quickly degraded by the NMD (Goyenvalle et al, 2012b). This observation may be advantageous for treating dominant diseases by exon skipping.

*In vivo* gene transfer of AAV9-U7-AON-5+6 efficiently induced the skipping of exons 5 and 6 of *Mybpc3* and therefore increased Var-4 mRNA level as early as 7 days after injection (Fig 8). Var-4 expression was persistent for 22–25 days (Fig 5) and 55 days (Fig 9) post-injection in KI mice. These data provide evidence that AONs directed against ESEs of exon 5 and/or exon 6 are sufficient to produce a “therapeutic mRNA”. This is accompanied by markedly lower Mut-1 or Mut-1 plus Mut-3 mRNAs. It is well perceivable that efficient exon skipping replaced non-functional Mut-1/Mut-3 by Var-4 cMyBP-C protein. We also provide evidence that U7snRNA-mediated exon skipping rescued the cardiomyopathic phenotype in newborn KI mice 7 days after injection (Fig 7). The finding that AAV9-Var-4 systemic delivery similarly restored cardiac function ( Fig S7) supports the view that Var-4 is functional and non toxic for the heart, and at least in part, the reason for the functional rescue in KI mice. The rescue of the phenotype was much less obvious 55 days after administration of AAV9-U7-AON5 + 6 in newborn KI mice (Fig 9). On the one hand, AAV-dosing may play a role. This is supported by the observation of a 4-fold lower number of virus particles in the heart 55 days than 7 days after AAV9 injection ( Fig S6). The drop of virus particles in the first month after injection is in agreement with recent data (Hu & Lipshutz, 2012). This indicates the need to inject even a higher dose from the beginning or to inject later another dose of AAV. On the other hand, the different state of the diseased heart and developmental changes could also be relevant. It is apparent, for instance, that FAS went down over time in WT mice (from 53 to 43%), whereas it formally increased in KI mice between days 12–14 and 55–57 (Fig 9). The reasons are unknown at present, but it is apparent that LVM and FAS evolved similarly over time in AON-5+6-treated KI mice and WT (at a lower level) and clearly differed from PBS-treated KI. This in fact suggests a persistent therapeutic effect, which would be in line with the persistent molecular rescue. In addition, whereas 4-week-old KI mice showed marked hypertrophy (thickening of walls) in addition to LV dilation, newborn KI mice only exhibited LV dysfunction and dilation, and marked activation of *Nppa* and *Nppb* genes (Fig 6). Development of LVH started at postnatal days 3-4 and was associated with the activation of *Acta1* expression, but not of *Myh7*, which actually remains high in KI compared to WT (Fig 6). These findings are interesting as they indicate that dysfunction (systolic and likely also diastolic) precedes LVH in (this model of) HCM. Similar observations were made in 2 other mouse models of HCM (homozygous *Myh6*- and *Mybpc3*-targeted KI mice; Fatkin et al, 1999; McConnell et al, 1999), and in a human patient carrying a homozygous *MYBPC3* mutation, who died at the age of 9 month from a marked LV dilation and dysfunction (Richard et al, 2003).

In summary, this proof-of-principle study showed for the first time that AONs targeting ESEs in mouse *Mybpc3* induces the skipping of the corresponding exons and results in a modified in-frame mRNA and protein *in vitro* and *in vivo*. Importantly, systemic administration of AAV9 encoding U7-AON-5+6 was sufficient to abolish cardiac dysfunction and to prevent development of LVH in newborn KI mice. Although further optimization is needed to maintain the therapeutic cardiomyopathy rescue over an extended period, our findings paves the way for a causal therapy of HCM.

## MATERIALS AND METHODS

### *Mybpc3*-targeted knock-in mice

The investigation conforms to the NIH guidelines for the care and use of laboratory animals published by the NIH (Publication No. 85-23, revised 1985). The experimental procedures were in accordance with the German Law for the Protection of Animals and accepted by the Ministry of Science and Public Health of the City State of Hamburg, Germany (Nr. 69/10). Development and initial characterization of the *Mybpc3*-targeted KI mouse model was previously reported (Vignier et al, 2009). In brief, KI mice carry a G>A transition on the last nucleotide of exon 6, which was introduced by gene targeting using the Cre/lox system (Vignier et al, 2009). WT and KI mice were maintained on the Black Swiss genetic background.

### Culture and transfection of HEK293 cells

HEK293 cells were maintained at 37 °C and 7% CO_2_ in Dulbecco's modified Eagle's medium supplemented with 10% heat inactivated foetal bovine serum (Gibco) and 1% penicillin-streptomycin (Gibco). For transient transfections cells were plated at a density of 2.5 × 10^5^ cells in 12-well dishes and incubated until a confluency of 50–70%. Transfection with 2 µg plasmid DNA was conducted using TurboFect® (ThermoScientific) following the instructions of the manual. After 48 h cells were cultured for 15 min in the presence or absence of forskolin (10 µM) and 3-Isobutyl-1-methylxanthine (IBMX, 250 µM), which increases cAMP level and therefore activate PKA. DMSO was used as a negative control. Cells were then harvested for RNA and protein analysis.

### AON design

AON-5 (5′-CCA GCC ACU CGG GCU GAG AAG ACA A-3′) and AON-6 (5′-AAG UGG UCU GAG CAU CUG UGA UGU G-3′) target ESE motifs in exon 5 or exon 6 of *Mybpc3*, respectively. The motifs were identified using the ESE prediction program “ESE finder 3.0” (Cartegni et al, 2003; Smith et al, 2006). To test the specificity of the AONs (25-mers) 2′-*O*-methyl RNA with a full-length phosphorothioate 2OMePS backbone was synthesized (Eurogentec).

### Culture and transfection of neonatal mouse cardiac myocytes

NMCMs were isolated from up to 25 hearts of 0-4-day-old pups and cultured as described previously (Vignier et al, 2009). After 3 to 4 days of plating cytosine arabinoside (Ara-C) was added (25 µM) to prevent division of remaining fibroblast cells. WT or KI NMCMs were transfected with 5 µg modified AON-5 or AON-5+6. The most effective AON quantity was determined in a pre-test. TurboFect™ was used for all transfections according to the manufacturer instructions. Non-transfected, but transfection reagent-containing (mock) NMCMs served as controls.

### Production and transduction of adeno-associated viral vectors *in vitro* and *in vivo*

For AAV production the self-complementary vector pAAVscU7 and the HEK293-AAV cell line (Cell Biolabs) were used. Cells were maintained at 37 °C and 5% CO_2_ in Dulbecco's modified Eagle's medium supplemented with 10% heat inactivated foetal bovine serum (Gibco) and 1% penicillin-streptomycin (Gibco). Both AON-5 and AON-6 were inserted in separated cassettes each containing the modified U7snRNA gene along with its natural promoter and 3′ elements. Both cassettes were then cloned in tandem into the *Xba*I and *Avr*II sites in pAAVscU7. For production of AAV6 pseudotyped vectors a double transfection of pAAVscU7-AON-5+6 snRNA and pDP6rs (kindly provided by J. Kleinschmidt, Heidelberg) encoding adenovirus helper functions, AAV2 rep and AAV6 cap genes in HEK293T cells were conducted (Grimm et al, 2003; Muller et al, 2006). AAV9 pseudotyped vectors were prepared by triple-transfection of pAAVscU7-AON-5+6 snRNA, pXX6 encoding adenovirus helper functions and pAAV2-9 (RepCap) in HEK293 cells. Vector particles were purified 72 h after transfection on iodixanol gradients from cell lysates. Gradient fractions were concentrated using centrifugal cartridges (Amicon ultra-4 50K or ultra-15 100 K), whereby iodixanol was exchanged against PBS or PBS-MK (Gibco; supplemented with 1 mM MgCl_2_ and 2.5 mM KCl). AAV6 titers were determined in triplicates by qPCR using the Maxima™ SYBR Green/ROX qPCR Master Mix (ThermoScientific) and primers complementary to vector sequences (forward: 5′-ACT CGG GCT GAG AAG ACA AAA TTT TTG GAG CA-3′; reverse: 5′-GAG TGG CCA GGC GAG GAG-3′). AAV9 titers were determined in triplicates by qPCR using a Taqman-specific probe (forward: 5′-CTC CAT CAC TAG GGG TTC CTT-3′; reverse: 5′-GTA GAT AAG TAG CAT GGC-3′; probe: 3-TAG TTA ATG ATT AAC CC-MGB-3′). Prior to titration viral DNA was extracted by diluting the virus 1:10 in TE buffer (20 µl total; 1 mM Tris–HCl pH 8, 0.01 mM EDTA), addition of 20 µl 2 M NaOH, incubation at 56 °C for 30 min and neutralization with 960 µl 40 mM HCl. The viral genome copy numbers (vg) were calculated by normalization of the virus-DNA dilutions to the corresponding plasmid-DNA dilutions (plasmid standard curve). Titrations were performed on the ABI PRISM® 7900HT Sequence Detection System (Applied Biosystems).

NMCMs were transduced with AAV6 (1 × 10^5^ vg) 30 min prior plating at room temperature. As a control for transduction efficiency AAV6 encoding GFP gene was used. Analyses were done 48 h after transduction. Four-week-old mice received 9.4 × 10^11^ vg of AAV9-U7-AON5 + 6 in a total volume of 150 µl by tail-vein injection using 27 and 29 gauge needles. Control animals received 150 µl of AAV9-GFP (7.6 × 10^10^ vg), PBS or NaCl. The mice recovered quickly from the injection without loss of mobility. Administration of AAV9 in 1-2-day-old mice was performed under hypothermia by injection into the vena temporalis superficialis using 30 gauge needles (Sands & Barker, 1999). Five KI mice received 2 × 10^11^ vg of AAV9-U7-AON5 + 6 in a total volume of 50 µl and control animals (WT and KI) received 50 µl PBS.

### mRNA analysis

Total RNA was extracted from cultured NMCMs or mouse ventricles (30 mg) using the SV Total RNA kit (Promega) or Trizol® (Invitrogen), according to the manufacturers' instructions. RNA concentration, purity and quality were analysed using the NanoDrop® ND-1000 spectrophotometer (Thermo Scientific). Reverse transcription was performed from 100–200 ng RNA using oligo-dT primers (SuperScript™ III kit, Life Technologies). The quality of cDNA was validated by PCR using AmpliTaq Gold® (Applied Biosystems) and murine GAPDH primers (forward: 5′-ATT CAA CGG CAC AGT CAA G-3′; reverse: 5′-TGG CTC CAC CCT TCA AGT-3′). Touch-down PCR (65 to 60 °C) using AmpliTaq Gold® polymerase according to the manufacturer's instructions was carried out in a total volume of 20 µl for 35-cycles with a forward primer located in exon 4 (5′-TCT TTC TGA TGC GAC CAC AG-3′) and a reverse primer located in exon 9 (5′-TCC AGA GTC CCA GCA TCT TC-3′). For validation of the alternative spliced Var-4 isoform in WT NMCMs or ventricular tissue, two rounds of PCR were conducted using primers complementary to exon 4 (5′-TCT CGG TAA CCC AGG ATG G-3′) and exon 7 (5′-GCT GAT CTG AGG TCC AGG TCT-3′) or exon 9 (5′- TCC AGA GTC CCA GCA TCT TC -3′). The PCR products were visualized on a 2% agarose gel. Subsequent gel extraction using the QIAquick Gel Extraction Kit (Qiagen) and sequencing (MWG) were performed. The list of all primers used is available on request.

### Western blot analysis

Crude protein extract was obtained from 50 mg ventricular tissue or from one well (12-well-plate) of cultured NMCMs or HEK293 cells, which was rinsed once with ice-cold 1x D-PBS (Life Technologies). Proteins were extracted in lysis buffer (30 mM Tris base pH 8.8, 5 mM EDTA, 30 mM NaF, 3% SDS, 10% glycerol). The tissue was homogenized with 5 volumes of lysis buffer using the Tissue Lyser (Qiagen; 2 × 30 s at 30 Hz) and the protein homogenate centrifuged at 13,200 rpm for 10 min at room temperature. The supernatants were collected and protein concentration determined using the Bradford protein assay (BioRad). Some samples were extracted with Trizol® (Invitrogen) followed by pellet solubilization in 8 M Urea in 50 mM Tris, pH 7.4. Proteins (HEK293 cells: 0.5–30 µg; NMCMs: 35–50 µg and murine tissues: 7.5–15 µg) were either loaded on 6–10% acrylamide/bisacrylamide (29:1) gels or 4–12% NuPAGE® Bis–Tris gels (Life Technologies) and electrotransferred on nitrocellulose membranes (Whatman®). Membranes were incubated overnight with primary antibodies (polyclonal antibodies directed against Ser-282-cMyBP-C (1:1000; custom made; El-Armouche et al, 2007), the MyBP-C motif of cMyBP-C (1:1000; gift from C. Witt, Mannheim, Germany), 2-14aa- (1:15,000), Ser273- (1:2000) and Ser-302-cMyBP-C (1:10,000; gifts from S. Sadayappan, Chicago, IL); Var-4-cMyBP-C (1:1000; custom made), or total ERK (p44/42 MAPK; 1:1000; Cell Signaling); monoclonal antibodies directed against α-actinin (1:1000, Sigma) or the FLAG epitope (1:5000, Sigma). The membranes were washed and incubated with peroxidase-labeled secondary antibodies (anti-rabbit IgG 1:5000, Dianova or anti-rabbit IgG 1:6000, Sigma). Protein bands were visualized using the SuperSignal® West Dura (Pierce) according to manufacturer's instructions. Signals were detected with the Chemie Genius^2^ Bio Imaging System and quantified with the Gene Tool Software (Syngene, Cambridge). Obtained values were plotted using the software GraphPad Prism 5 (GraphPad Software, Inc.).

### Immunofluorescence analysis

AAV6-transduced KI-NMCMs were cultured for 48 h on coverslips. Cells were rinsed with 1x D-PBS and fixed in methanol/acetone (20/80; 10 min at −20°C). After rinsing in PBS (2 × 5 min), cells were permeabilized for 1 h at room temperature in solution A (10% FCS, 1% BSA, 0.5% Triton X-100 in PBS) followed by another washing step in solution B (1% BSA, 0.5% Triton X-100 in PBS). Cells were incubated for 1 h at room temperature with the primary antibodies diluted in solution B (monoclonal anti-FLAG (1:800, Sigma); polyclonal anti-cMyBP-C-2-14aa (1:10,000; gift of S. Sadayappan, Chicago, IL) and anti-titin-Z1 (1:200; gift of S. Labeit, Heidelberg, Germany). Cells were rinsed twice in solution B and incubated for 1 h at room temperature with secondary antibodies diluted in solution B (anti-rabbit IgG Alexa 546-conjugated, 1:800 and anti-mouse IgG Alexa 488-conjugated, 1:800). After nuclear staining with TO-PRO-3 (Molecular Probes), cells were rinsed in PBS and embedded in Mowiol. Analysis was performed by confocal microscopy using a Zeiss Axiovert microscope with a 63×-oil objective. Confocal images were recorded with a Zeiss LSM 710 system.

**The paper explained**

PROBLEM:

Exon skipping mediated by AONs is a promising therapeutic approach for selected genetic disorders, but has not yet been evaluated for cardiac genetic diseases. Hypertrophic cardiomyopathy (HCM) is often caused by mutations in *MYBPC3* encoding cardiac myosin-binding protein C. The study investigated the feasibility and efficacy of viral-mediated AON transfer in a *Mybpc3*-targeted knock-in (KI) mouse model of HCM.

RESULTS:

KI mice carry a homozygous G>A transition in exon 6, which results in 3 different aberrant mRNAs and/or proteins. In addition, we identified an alternative variant (Var-4) deleted of exons 5–6 in wild-type and KI mice. To enhance its expression and suppress aberrant mRNAs we designed AON-5 and AON-6 that mask ESE motifs in exons 5 and 6. AONs were inserted into modified U7 small nuclear RNA and packaged in adeno-associated viral vectors (AAV-U7-AON-5+6). Treatment with AAV-U7-AON-5+6 markedly increased Var-4 mRNA and protein levels and reduced aberrant mRNAs. Systemic administration in newborn KI mice restored cardiac function and prevented left ventricular hypertrophy.

IMPACT:

The present study provides the first proof-of-principle evidence that AAV-U7-AONs remove a mutation in neonatal mouse cardiac myocytes and *in vivo* in the heart of a HCM mouse model. AON-mediated exon-skipping increased the amount of the internally deleted but functional cMyBP-C variant. Cardiomyopathy was rescued through U7snRNA-mediated exon skipping in neonatal mice. Therefore, our findings laid the first stone towards a causal therapy of HCM.

### Echocardiography

Transthoracic echocardiography was performed using the Vevo 2100 System (VisualSonics, Toronto, Canada). Animals were anaesthetized with isoflurane (1–2%) and assured to a warming platform in a supine position. B-mode images were obtained using a MS400 transducer for adult mice and a MS550 transducer for neonatal mice. For 4-week-old mice echocardiography was performed prior tail-vein injection and then weekly, and for newborn mice 7 days after injection. Images were obtained in a parasternal short and long axis view. The dimensions of the left ventricle (thickness of the septum and posterior wall, as well as left ventricular diameter) were measured in a short axis view in diastole and systole.

### Statistical analysis

Data were expressed as mean ± SEM. Statistical analyses were performed using the unpaired Student's *t*-test or the one-way or two-way ANOVA followed by Dunnett or Bonferroni post-*hoc* test using the commercial software GraphPad Prism5 (Software Inc.). A value of *p* < 0.05 was considered statistically significant.

## For more information

DZHK (Deutsches Zentrum für Herz-Kreislauf-Forschung e.v., DZHK): German Centre for Cardiovascular Research http://dzhk.de/

BIG-Heart (Health-F2-2009-241577): European network on hypertrophic cardiomyopathy http://www.big-heart.eu/

Genethon: Biotherapies Institute http://www.genethon.fr/en/

Institut de Myologie http://www.institut-myologie.org/

## Supplementary material

As a service to our authors and readers, this journal provides supporting information supplied by the authors. Such materials are peer reviewed and may be re-organized for online delivery, but are not copy-edited or typeset. Technical support issues arising from supporting information (other than missing files) should be addressed to the authors.
