# Supplementary material for: Rescue of cardiomyopathy through U7snRNA-mediated exon skipping in Mybpc3-targeted knock-in mice
Source: EMBO Mol Med. 2013 May 29;5(7):1060–77. doi: 10.1002/emmm.201202168 (PMC3721478; doi:10.1002/emmm.201202168)
Supplement: Supplementary file 1 [file emmm0005-1060-SD1.pdf]

EMBO Molecular Medicine

Rescue of cardiomyopathy through U7snRNA-mediated exon skipping in Mybpc3-targeted knock-in mice

Christina Gedicke-Hornun, Verena Behrens-Gawlik, Silke Reischmann, Birgit Geertz, Doreen Stimpel, Florian Weinberger, Saskia Schlossarek, Guillaume Précigout, Ingke Braren, Thomas Eschenhagen, Giulia Mearini, Stéphanie Lorain, Thomas Voit, Patrick A. Dreyfus, Luis Garcia, Lucie Carrier

Corresponding author: Lucie Carrier, University Medical Center Hamburg-Eppendorf

Review timeline:

Submission date:	19 October 2012
Editorial Decision:	27 November 2012
Revision received:	26 March 2013
Editorial Decision:	18 April 2013
Revision received:	19 April 2013
Accepted	19 April 2013

Transaction Report:

(Note: With the exception of the correction of typographical or spelling errors that could be a source of ambiguity, letters and reports are not edited. The original formatting of letters and referee reports may not be reflected in this compilation.)

Editor: Natascha Bushati / Céline Carret

1st Editorial Decision 27 November 2012

Thank you for the submission of your manuscript to EMBO Molecular Medicine. We have now heard back from the three referees whom we asked to evaluate your manuscript. As you will see from the reports below, the referees find the topic of your study of potential interest. However, they raise substantial concerns on your work, which would have to be convincingly addressed in a major revision of the present manuscript.

Reviewer #1 highlights that detailed quantification of Mybpc3 Var-4 in KI and wt mice and further analysis of the *in vivo* toxicity and functionality of Var-4 are needed to strengthen the study. This reviewer also requires in-depth characterisation of the neonatal phenotypes of KI mice.

Importantly, reviewers #2 and #3 feel that long-term analyses of the functional recovery of newborn AON-treated mice should be undertaken to substantiate the potential treatment approach.

Finally, reviewer #3 is concerned about the possible effects of Var-4 induced by AAV transduction and would like to see confocal analyses of Var-4 expression in wt, KI and AON-treated KI myocardia or adult myocytes.

Given the potential interest of your study, we would be willing to consider a revised manuscript with the understanding that the referees' concerns must be fully addressed.

Please note that it is EMBO Molecular Medicine policy to allow a single round of revision in order

to avoid the delayed publication of research finding. Consequently, acceptance or rejection of the manuscript will depend on the completeness of your responses included in the next version of the manuscript.

Revised manuscripts should be submitted within three months of a request for revision. If your revision will have to exceed this time frame, please contact the editor. Please also contact the editor as soon as possible if similar work is published elsewhere.

I look forward to seeing a revised form of your manuscript in due course.

***** Reviewer's comments *****

Referee #1 (General Remarks):

The manuscript by Gedicke-Hornung et al, describes the use viral-mediated transduction of antisense oligoribonucleotide (AON) to produce exon skipping in mice carrying a Mybpc3 missense mutation (denoted KI), a model for human hypertrophic cardiomyopathy (HCM). Prior studies had identified 3 abnormal Mybpc3 transcripts arise from this mutation. The author's report an additional variant (Var-4) that lacks Mybpc exons 5 and 6. They report that Var-4 is expressed at low-level expression in WT and KI mice, that it is phosphorylated, and incorporated into the sarcomere in neonatal mouse myocytes (NMCM). Adeno-associated virus (AAV) transcduction of AON was ineffective to rescue young adult KI mice, but corrected LV dilation and dysfunction in 1-day-old KI mice.

Main concerns:

1. More data is needed about Mybpc3 Var-4. Is this a strain-specific isoform? Is there expression in other rodents, mammals or humans?
2. What is the predicted function of sequences encoded by exon 5-6?

3. Levels of expression of Mybpc3 Var-4 appear to be quite low, as it was detected only after nested PCR. Provide levels of expression in vivo. Does its expression correlate with development, age, disease?
4. If a 20% increase in expression was achieved, it would be important to define the total change in Mybpc levels that would occur, as the mechanism of disease is due to inadequate protein.
5. Details are needed to demonstrated that Var-4 protein is functional and non-toxic in vivo. Over-expression of Var-4 in KI and WT mice would be useful.
6. Given that reduced levels of Mybpc causes HCM and evidence presented here that the AAV can transduce expression of Mybp3-var-4, discuss why the experimental design is not to transduce WT Mybp3 into HCM mice rather than increasing var-4 expression.

7. Page 5 discusses reasons why the AAV strategy failed to "cure" young adult KI mice. Given that GFP expression was detected for only 3-5 weeks after injection, it seems possible that the lack of improvement in LV parameter (Fig 5) might also occur because insufficient duration of AAV-mediated expression.

8. The discussion hypothesizes "that that AAV transduction is not effective in WT 48 h after treatment or that exon-skipped mRNAs are quickly degraded in WT cells by the NMD." Even for a proof-of-principle study, it is important to discriminate between these two (and other reasons) for the AAV transduction was ineffective in young adult KI mice.

9. The finding that 1-day-old KI mice exhibit LV dilation and dysfunction is surprising. Are there similar phenotypes in other mouse models of HCM or in the human disease. If not, why not?

10. Provide more details about this neonatal phenotypes: histopathology, ANP expression, etc.

11. It is critical to define the time course of the neonatal phenotype and the emergence of the HCM phenotype in order to demonstrate improvement of the neonatal phenotype by AAV.

12. Serial data (echo/histopathology/other markers of LV dysfunction) are needed to demonstrate

that AAV rescued neonatal KI mice.

Minor concerns:

1. Internal controls, such as GAPDH, are missing in Fig 4A, 5A. The authors should provide evidence to show that the same amount of RNA or mRNA was used.
2. ALl lanes in Fig4A, Fig 4B should included in one gel picture.
3. The levels of var-4 are inconsistent between in vitro cardiomyocytes (Fig4B) and in vivo KI mice (Fig 5B). As discussed above, the authors need to show levels of Var-4 mRNA or protein at different stages in KI and wild-type mice (such as embryonic, neonatal, pre-HCM, HCM stages)
4. Figure 4A appears to show that AON-5+6 reduced Mbpc3 Mut1 expression. Is this the mechanism by which AON-5+6 rescues the KI mice?
5. Figure 6: The authors need to provide change in mRNA and protein levels of Var-4 in neonatal mice rescue experiments.

Referee #2 (Comments on Novelty/Model System):

The KI mouse used in this study recapitulates features of the human disease adequately, but no correction is documented when young adult animals are treated. A therapeutic effect is observed when newborn animals are injected, but this situation is unlikely to mimic anything that will happen in the clinic. The stability of the correction is not documented, and this point is key to assess the validity of the approach anf the adequacy of the animal model

Referee #2 (General Remarks):

This paper describes a minor splice variant that skips exons 5 and 6 in the Mybpc3 gene. It is shown that the encoded protein is functional; suggesting that artificially induced skipping of these exons when they are mutated in patients with hypertrophic cardiomyopathy (HCM) could bring therapeutic benefit.
The authors show that antisense sequences, either in the form of synthetic oligonucleotides (AONs) or embedded in U7 snRNA chimeras can induce exon5 and 6 skipping in cultured cardiomyocytes and in HCM mice. Reversion of the HCM phenotype was seen when newborn animals were treated with an AAV vector, whereas young adults did not benefit from the treatment.
This is an interesting and well written paper that suggests the possibility of an exon skipping treatment for a subset of patients with HCM. The study presented here is however still preliminary with respect to a stable rescue of the diseased phenotype in the mouse model.

Specific comments:

1. The quantification of the different mRNA species needs to be explained (same for the protein levels on Western blots). Is it just by gel scanning, in which case one should not expect this to be very quantitative? Reproducibility of the RT PCR assay is challenged by the data shown in Fig S2 in which the ratio of the different species in the KI is inverted relative to Fig 1A and 4A. What is the explanation for this?
2. Given the data in Fig S2, it is curious that the authors did not try to use AON-5 alone in the AAV construct, because it seems to result in only one Var-4 coding band, rather than a mixture with Mut1-3 bands when both AON 5 and 6 are used. Please explain the rationale for this.
3. In Fig 5B, the quantitation of Var 4 is again problematic. What do these numbers really represent since they are obtained with an antibody "specific" for variant 4? Is the total amount of protein calculated using the background signal for the full length protein?
4. At least an RT PCR analysis of Mybpc3 transcripts in the heart after AAV transduction is needed to evaluate how much of the effect - or absence of effect- is related to skipping efficiency.
5. It is very disappointing not to have data on a couple of animals injected as newborns and kept for a few months. Seven days is really short term, even to reach a steady state level of AAV mediated gene expression. Long term data would help evaluating the relevance of the approach. Are the AS sequences still present and expressed once the heart is fully developed?
6. P6, discussion: "It is well perceivable that efficient exon skipping at this early time point rescues dysfunction by restoring normal cMyBP-C protein levels" Where are those "normal levels"

documented in the paper?

Referee #3 (Comments on Novelty/Model System):

AAV9 mediated delivery of AONs in a knockin mouse model is already optimal and would be the choice for human therapy of this cardiac disease. The technical execution of the study and its model leaves a bit to be desired.

Referee #3 (General Remarks):

Gedicke-Hornung et al. Rescue of cardiomyopathy through U7snRNA-mediated exon skipping in Mybpc3 -targeted knock-in mice

Exon skipping mediated by antisense oligoribonucleotides (AON) is promising, but has not yet been evaluated for cardiac genetic diseases. Using a Mybpc3 targeted knock-in (KI) mouse model of hypertrophic cardiomyopathy (HCM), carry a homozygous G>A transition in exon 6, adeno-associated virus-mediated delivery of AONs was tested that evoke exon-skipping resulted in an expected Mybpc3 splice isoform (Var-4) and abolished cardiac dysfunction and prevented left ventricular hypertrophy in newborn mice but failed to affect young adult mice. This study represents the very first description of a molecular therapy aimed to "genotypically" correct a serious and medically unmet common human disorder.

Major

Fig. 1A. Indication of the size on the size marker is lacking.

Fig. 2A. Indication of the size on the size marker is lacking.

Fig. 2B, C. Loading controls (e.g. tubulin, gapdh or the like) for the Western blots are lacking.

Fig. 2D, E. Although the immunofluorescence localization studies in (neonatal?) mouse KI myocytes are appreciated, questions that arise are to what extent the likely developmental and technical differences between cultured myocytes and the myocardium in situ are to be expected. Additionally, it was not clear to what extent the AAV transduction produces "supraphysiological" levels of Var-4, disrupting the "normal" pattern of Var-4. A more insightful analysis would include confocal analyses on intact myocardium WT, KI and AON-treated KI mice or adult myocytes.

Fig. 4A. Fig. 4A, B. Indication of the size on the size marker is lacking. Loading controls for the Western blots are lacking.

Fig. 4B indicates that an AAV6 was used for a subset of experiments, but Fig. S3 depicts characteristics of an AAV serotype 9. Was either one a typo? If not, what differences in efficiency of exon skipping could exist between AAV6 and AAV9?

Fig. 5A. Indication of the size on the size marker is lacking.

The most intriguing part of the study includes the phenotypic differences between adult vs newborn AON treated mice (Fig.5C and Fig.6B), where neonatal mice displayed benefit from exon-skipping but young adults failed to display phenotypic benefit despite more or less equally efficient exon-skipping. The conclusions - difference between neonatal and young adult mice - seem preliminary. The authors should first exclude that differences in escalating doses of AAV could reach a threshold of efficiency of exon skipping after which phenotypic benefit occurs, or, alternatively, waiting for more prolonged time periods could yield different results. One straightforward analysis that is required is whether the functional benefit recorded in 1 day-old mice (Fig. 6) is maintained throughout their life-span or at least well into adulthood.

1st Revision - authors' response 26 March 2013

Response to Reviewer 1

We would like to thank the reviewer for his/her careful work on our manuscript and his/her nice comments. We have carefully considered all of the suggestions and performed extensive new experiments. We feel that our manuscript has been substantially improved. Please note that all changes in the manuscript are marked with yellow.

Referee #1 (General Remarks):

The manuscript by Gedicke-Hornung et al, describes the use viral-mediated transduction of antisense oligoribonucleotide (AON) to produce exon skipping in mice carrying a Mybpc3 missense mutation (denoted KI), a model for human hypertrophic cardiomyopathy (HCM). Prior studies had identified 3 abnormal Mybpc3 transcripts arise from this mutation. The author's report an additional variant (Var-4) that lacks Mybpc exons 5 and 6. They report that Var-4 is expressed at low-level expression in WT and KI mice, that it is phosphorylated, and incorporated into the sarcomere in neonatal mouse myocytes (NMCM). Adeno-associated virus (AAV) transcduction of AON was ineffective to rescue young adult KI mice, but corrected LV dilation and dysfunction in 1-day-old KI mice.

Main concerns:

1. More data is needed about Mybpc3 Var-4. Is this a strain-specific isoform? Is there expression in other rodents, mammals or humans?

We first evaluated whether Var-4 is expressed in different mouse genetic backgrounds and whether its expression varies with development. Our results show:

1) Var-4 is not a strain-specific isoform: Var-4 is expressed in cardiac myocytes isolated from both Black swiss and C57BL/6J neonatal mice (new Fig S2A of Supporting information)
2) Var-4 is expressed at low level in the mouse heart during the entire development of either Black swiss or C57BL/6J mice. There was no major difference in the pattern of Var-4 expression between embryonic, neonatal, adult, and old mice (new Fig S2 of Supporting Information).

These data are now included in the new Fig S2 of the Supporting information, and presented in the Results (page 4, lines 16-18) and Discussion (page 6, lines 39-40).

We evaluated whether Var-4 is detected in normal and diseased human hearts. However, and even after 2 rounds of PCR, Var-4 was not detected in human, particularly in HCM septal myectomy from patients carrying the same mutation as the KI mice at the heterozygous state, which also results in Mut-2 mRNA (Figure not included in the revised version).

2. What is the predicted function of sequences encoded by exon 5-6?

Exons 5 and 6 encode a major part of the C1 domain of cMyBP-C, which is a well-established pattern for Immunoglobulin-like I (IgI) domain. The phosphorylation sites of cMyBP-C, which plays an important role in cardiac function are not located in the C1 domain, but in the MyBP-C motif. We show in the present study that these sites remain and can be phosphorylated *in vitro* after transfection of HEK cells with FLAG-variant-4 cDNA (Fig 2C).

3. Levels of expression of Mybpc3 Var-4 appear to be quite low, as it was detected only after nested PCR. Provide levels of expression in vivo. Does its expression correlate with development, age, disease?

Please see the answer to your question 1. The level of Var-4 mRNA is very low in neonatal cardiac myocytes and in the heart in wild-type mice and did not differ between different stages of the development. In addition, Var-4 mRNA level did not differ in ventricular tissue of young or old KI mice (New Fig S2C of Supporting Information, and Results page 4, lines 16-18):

4. If a 20% increase in expression was achieved, it would be important to define the total change in Mybpc levels that would occur, as the mechanism of disease is due to inadequate protein.

The reviewer is right, although evidence for a poison peptide mechanism also exist. Anyhow, we did different experiments:

1. We repeated the Western blot of Fig 5B (AAV9 administration in 4-wk-old mice, evaluation 3-5 wks after) with two different antibodies: The left panel shows the membrane stained with the anti-cMyBP-C antibody and the right panel the other part of the membrane stained with the anti-Var-4 antibody. Now we see the same results with both antibodies. Again Var-4 antibody recognizes both Var-4 and (after transfection in HEK293 cells) wild-type cMyBP-C (in WT mice), indicating that it is not specific for Var-4. In these conditions, there was no major difference in the level of total cMyBP-C between the groups, and, when compared to WT samples, still a low level of cMyBP-C (about 20-30%). In addition, the level of Var-4 was less than in the previous experiment. We therefore removed the values from the blot in the revised version of the manuscript. Treatment in 4-wk-old mice did not result in a marked accumulation of Var-4 protein.

2. We also evaluated the amount of Var-4 and total cMyBP-C 7 days and 55 days after AAV9-U7-AON-5+6 administration in newborn mice (New Fig 8 and new Fig 9, respectively). In both cases, the amount of total cMyBP-C increased after AON treatment, although it did not reach the level found in the WT hearts. However, we now show the shift in isoform composition (more Var-4 and less/absence of Mut-1/Mut-3) with improved cardiac function (Fig 8A). This suggests that Mut-1/Mut-3 may be dominant-negative isoforms. After 7 days (Fig 8A) and after 55 days (Fig 9C):

5. Details are needed to demonstrate that Var-4 protein is functional and non-toxic in vivo. Over-expression of Var-4 in KI and WT mice would be useful.

Thank you for suggesting this. We developed an AAV9-FLAG-Var-4 under the control of the CMV promoter. 1-day-old KI mice received either $2x10^{11}$ vg of this AAV9 or same volume of PBS via the temporal vein. Functional and molecular analyses were done 7 days thereafter. These data showed that the expression of Var-4 i) prevents left ventricular hypertrophy and accumulation of the hypertrophic marker *Acta1* mRNA, and ii) restores fractional area shortening and levels of *Nppa* and *Nppb* to normal.

These data suggest that Var-4 is functional and not toxic in vivo and supports the exon skipping strategy using the AON-5+6 in vivo to rescue the phenotype of KI mice.

These data are included in Fig S7 of Supporting information, in the Results (page 6, lines 5-10) and in the Discussion (page 7, lines 27-30).

6. Given that reduced levels of Mybpc causes HCM and evidence presented here that the AAV can transduce expression of Mybp3-var-4, discuss why the experimental design is not to transduce WT Mybp3 into HCM mice rather than increasing var-4 expression.

Thank you for your comment. There are indeed two other ways to produce full-length mRNA and protein, which are the spliceosome-mediated RNA trans-splicing and gene therapy. These strategies are also evaluated in our lab, but both strategies are beyond the scope of the present manuscript.

7. Page 5 discusses reasons why the AAV strategy failed to "cure" young adult KI mice. Given that GFP expression was detected for only 3-5 weeks after injection, it seems possible that the lack of improvement in LV parameter (Fig 5) might also occur because insufficient duration of AAV-mediated expression.

This is possible, we could not exclude this. On the other hand, GFP expression shown in Fig S5A of Supporting Information was also evaluated 4 weeks after AAV9 administration, time at which the mice were evaluated by echocardiography in Fig 5.

We performed another experiment to evaluate the efficiency of transduction *in vivo*. 1-day-old mice received either AAV9-CMV-GFP ($2x10^{11}$ vg) or PBS for 7 days. Cardiac sections were performed and analysed by immunofluorescence. The data showed GFP staining only in the mouse that received GFP but not in the other, and the transduction efficiency is very high (new Fig S5B of Supporting information):

Finally, we also evaluated the pattern of *Mybpc3* mRNAs 7 days or 55 days after systemic administration of AAV9-U7-AON5+6. After 7 days, we already see that AON5+6 stabilized the Var-4 mRNA (and markedly reduced the level of Mut-1/Mut-3 mRNA; New Fig 8):

After 55 days, the amount of Var-4 was still higher in ventricular tissue of KI mice injected with AAV9-U7-AON5+6 than with PBS, and Mut-1 mRNA was still lower than in KI-PBS (new Fig 9B):

We also evaluated the quantity of AAV9 virus genome particles in the ventricular tissue of neonatal KI mice injected for different times with 2x10E+11 vg (mean dose of $1.44x10^{14}$ vg/kg body weight) of AAV9-U7-AON5+6. The results show that the amount of AAV9 particles decreased 5-fold in mouse ventricular tissue between 7-day- to 28-day treatments (new Fig S8 of Supporting information):

Similarly, we analysed the amount of particles in ventricular tissue from 4 wk-old KI mice injected with 9.4x1011 vg (mean dose $4.8x10^{13}$ vg/kg BW). The amount of AAV9 particles in the ventricular tissue was also 5-fold lower than after 7 day-treatment in neonates (not shown).

These findings indicate that:

i) the AAV9 particles are still present 55 days post-injection but to a 4-fold lesser extent than 7 days post-injection; this is in agreement with previous studies showing a 10-fold decrease in the virus particles 1 month after injection in vivo (Hu and Lipshutz Gene Therapy 2012);

ii) the exon skipping was demonstrated 7 days after and 55 days after AAV9 administration; Therefore, the 4-wk time evaluation in young adult should have been enough to see the effect;

iii) the phenotype was fully rescued when the virus particles are elevated (7 days), but the rescue was much less obvious when the virus particles was reduced (55 days); we therefore believe that the AAV9 dose injected in 4-wk-old mice was too low to see the functional rescue (virus particles 3-fold lower than in neonates);

8. The discussion hypothesizes "that that AAV transduction is not effective in WT 48 h after treatment or that exon-skipped mRNAs are quickly degraded in WT cells by the NMD." Even for a proof-of-principle study, it is important to discriminate between these two (and other reasons) for the AAV transduction was ineffective in young adult KI mice.

Thank you very much for your suggestion. We evaluated whether the NMD is involved in WT cells by using the translation inhibitor emetine, which should stabilise the nonsense mRNAs. Cardiac myocytes were isolated from wild-type mice and transduced with AAV6-U7-AON5 or AAV6-U7-AON5+6 (MOI 10,000) for 72 h. Figure below shows:

i) detection of Var-4 (even low) after 72 h in the WT cells

ii) Emetine treatment (300 µg/ml for 4 h) revealed the presence of mRNA deleted of exon 5 after AAV6-U7-AON5 and the presence of mRNAs deleted of either exon 5 or exon 6 (in addition to the expected Var-4).

These data suggest that 72 h transduction with AAV6 was finally sufficient to see Var-4 mRNA in WT myocytes and that the other nonsense mRNAs are degraded by the NMD. These data are integrated in the Fig S6 of Supporting information and in the Results (page 5, lines 1-5) and the Discussion has been modified accordingly (page 6, lines 14-19).

9. The finding that 1-day-old KI mice exhibit LV dilation and dysfunction is surprising. Are there similar phenotypes in other mouse models of HCM or in the human disease. If not, why not?

Yes, similar phenotype was observed in the 2 other HCM targeted KI mice: i) *Myh6*-targeted KI mouse model: although no phenotype was detected at day 0, it shows higher LVDD and LVSD than WT at day 4, followed by LV dysfunction and LVH at day 6 (Fatkin et al., JCI 1999); ii) in another *Mybpc3*-targetd KI mice (McConnell et al., JCI 1999), the phenotype started in the first 3 days postnatal with higher BW, LVDD and LVSD, and reduced LVFS than WT mice. This is also the case in a human HCM patient carrying a homozygous truncating *MYBPC3* mutation, who died at 9 month of age with marked LV dilation and cardiac dysfunction (Richard et al., Circulation 2003). These papers are now cited in the Discussion (page).

10. Provide more details about this neonatal phenotype: histopathology, ANP expression, etc.

We now provide evidence that LV dilation and dysfunction (Figure 6a) are associated with increased *Nppa* and *Nppb* mRNA levels, but not with *Myh7* and *Acta1* mRNA in 1-day-old KI mice (new Fig 6B):

Furthermore, the absence of activation of *Acta1* and *Myh7* at day 1 suggested us to evaluate the HW/BW and the pattern of the fetal gene program between day 1 and day 7 in both WT and KI mice. The results were really interesting. At day 1, only *Nppa* and *Nppb* are up-regulated in KI mice. At day 2, *Acta1* starts to be up-regulated (new Fig 6C):

11. It is critical to define the time course of the neonatal phenotype and the emergence of the HCM phenotype in order to demonstrate improvement of the neonatal phenotype by AAV.

Please see response to comment 10.

12. Serial data (echo/histopathology/other markers of LV dysfunction) are needed to demonstrate that AAV rescued neonatal KI mice.

Please see response to previous comments. We now provide more data in neonatal mice (Fig 6, Fig 7, Fig 8 and Fig S7.

Minor concerns:

1. Internal controls, such as GAPDH, are missing in Fig 4A, 5A. The authors should provide evidence to show that the same amount of RNA or mRNA was used.

Thank you for your suggestion. However, we believe that we do not need another internal control in these figures, because the amount of Var-4 mRNA is expressed as percentage of the total *Mybpc3* mRNA, which represents 100%.

2. ALl lanes in Fig4A, Fig 4B should included in one gel picture.

Both experiments have been done again. In both cases, we also added samples that were transduced with AAV6-AON-5 alone (new Fig 4):

3. The levels of var-4 are inconsistent between in vitro cardiomyocytes (Fig4B) and in vivo KI mice (Fig 5B). As discussed above, the authors need to show levels of Var-4 mRNA or protein at different stages in KI and wild-type mice (such as embryonic, neonatal, pre-HCM, HCM stages)

We now evaluated Fig 5B with the same antibody as used in Fig 4B. Now we see that the level of Var-4 protein does not differ between neonatal cardiac myocytes and adult ventricular tissue (new Fig 5B).

We also evaluated the level of Var-4 mRNA at different stages of development of wild-type mice (please see responses to major comment 1.

For the KI mice, we do not see difference in the level of Var-4 mRNA with age (new Fig S2D):

4. Figure 4A appears to show that AON-5+6 reduced Mbpc3 Mut1 expression. Is this the mechanism by which AON-5+6 rescues the KI mice?

The mechanism by which AON-5+6 rescued the phenotype is not certain. However, and as you suggested, we now provide new data showing the complete disappearance of Mut-1/Mut-3 mRNA in treated mice (New Fig 8A). These data suggest that Mut-1/Mut-3 plays a dominant-negative effect on the function. This is now also discussed in the revised version (page 7).

© EMBO

5. Figure 6: The authors need to provide change in mRNA and protein levels of Var-4 in neonatal mice rescue experiments.

Done (new Fig 8).

Response to Reviewer 2

We would like to thank the reviewer for his/her careful work on our manuscript and the critique. We have performed new experiments to address your points.

Please note that all changes in the manuscript are marked with yellow.

Referee #2 (Comments on Novelty/Model System):

The KI mouse used in this study recapitulates features of the human disease adequately, but no correction is documented when young adult animals are treated. A therapeutic effect is observed when newborn animals are injected, but this situation is unlikely to mimic anything that will happen in the clinic. The stability of the correction is not documented, and this point is key to assess the validity of the approach and the adequacy of the animal model

Referee #2 (General Remarks):

This paper describes a minor splice variant that skips exons 5 and 6 in the Mybpc3 gene. It is shown that the encoded protein is functional; suggesting that artificially induced skipping of these exons when they are mutated in patients with hypertrophic cardiomyopathy (HCM) could bring therapeutic benefit.

The authors show that antisense sequences, either in the form of synthetic oligonucleotides (AONs) or embedded in U7 snRNA chimeras can induce exon5 and 6 skipping in cultured cardiomyocytes and in HCM mice. Reversion of the HCM phenotype was seen when newborn animals were treated with an AAV vector, whereas young adults did not benefit from the treatment.

This is an interesting and well-written paper that suggests the possibility of an exon skipping treatment for a subset of patients with HCM. The study presented here is however still preliminary with respect to a stable rescue of the diseased phenotype in the mouse model.

Specific comments:

1. The quantification of the different mRNA species needs to be explained (same for the protein levels on Western blots). Is it just by gel scanning, in which case one should not expect this to be very quantitative? Reproducibility of the RT PCR assay is challenged by the data shown in Fig S2 in which the ratio of the different species in the KI is inverted relative to Fig 1A and 4A. What is the explanation for this?

Yes, the amount of Var-4 mRNA or the amount of Var-4 protein is expressed as a percentage of the total (representing 100%). This is now better explained in the legends of the Figures.

Yes, you are right that there is variability in the amount of mutant mRNA between old Fig S2 (new Fig S3) and Figs 1A and 4A. In order to see whether this was an artefact or related to the different culture times (long in Fig S2 and short in Figs 1A and 4A), we performed a novel experiment, in which we evaluated the pattern of mutant *Mybpc3* mRNA with culture time of KI NMCMs. These data showed that increasing time of culture stabilized mutant-2 over total *Mybpc3* mRNA. The

mechanism is not known and may involve inhibition of the NMD by substances contained in culture medium (Figure not included in the revised version).

2. Given the data in Fig S2, it is curious that the authors did not try to use AON-5 alone in the AAV construct, because it seems to result in only one Var-4 coding band, rather than a mixture with Mut1-3 bands when both AON 5 and 6 are used. Please explain the rationale for this.

The reviewer is right and actually based on these data using modified AONs, we also produce an AAV6-AON5 as well. Unexpectedly, the effects were less efficient than with modified AON5 in NMCM (see new Fig 4) and in rat engineered heart tissue (see Figure below of RT-PCR analysis using primers located in exons 4 and 9 of *Mybpc3*; not included in the revised version). This is the reason why we continued with AAV9-AON5+6 for the *in vivo* analyses.

3. In Fig 5B, the quantitation of Var 4 is again problematic. What do these numbers really represent since they are obtained with an antibody "specific" for variant 4? Is the total amount of protein calculated using the background signal for the full-length protein?

The antibody has been designed to recognize the aminoacid sequence resulting from the skipping of exons 5 and 6, and therefore the junction between Exon 4 and Exon 7. However, it also recognizes the WT or mutant-1 or mutant-3 isoforms.

We now did again the Western blot with 2 different antibodies and the Figure 5B has been made new accordingly. We also removed the percentage of Var-4 protein, because it was underestimated by calculating only the results obtained with the Var-4 antibody.

4. At least an RT PCR analysis of Mybpc3 transcripts in the heart after AAV transduction is needed to evaluate how much of the effect - or absence of effect- is related to skipping efficiency.

Done (new Fig 8). We evaluated the level of *Mybpc3* transcript and the cMyBP-C protein 7 days after AAV9 U7-AON-5+6 administration in newborn KI mice. The data showed AON-5+6 induced accumulation of Var-4 mRNA and protein and decreased in Mut-1/Mut-3 mRNA and proteins:

5. It is very disappointing not to have data on a couple of animals injected as new-borns and kept for a few months. Seven days is really short term, even to reach a steady state level of AAV mediated gene expression. Long-term data would help evaluating the relevance of the approach. Are the AS sequences still present and expressed once the heart is fully developed?

We thank the reviewer for his/her suggestion. We now performed the experiments.

1) First, we evaluated functional effects over a 55-day period.

Although we saw a complete rescue 7-15 days after administration of the AAV9-U7snRNA-AON5+6 *in vivo*, the rescue of the cardiac phenotype was not obvious anymore 55 days after treatment (new Fig 8). It should be noted, though, that the functional data in these young mice (to the best of our knowledge not systematically studied so far) are not easy to interpret. WT animals showed a marked reduction in FAS over time and this was the same, albeit at a lower level, in AAV9-injected KI mice. In contrast, FAS remained reduced in KI. From the similar pattern of WT and AAV-KI, the data can as well be interpreted as showing that the therapy was in fact effective even for the extended period. We have discussed this issue in the revised version.

We also evaluated and compared the amount of AAV9 particles in ventricular tissue of mice 7 days or 55 days after AAV9 administration. This shows that the virus genomes decreased by a factor of 4 with time (new Fig S6 of Supporting Information):

Despite the decrease in AAV9 particles with time, we observed a persistent expression of Var-4 in AAV9-treated mice (new Fig 9). However, Mut-1 mRNA was still detected but its level was 2-fold lower in AAV9-U7-AON-5+6 injected mice:

6. P6, discussion: "It is well perceivable that efficient exon skipping at this early time point rescues dysfunction by restoring normal cMyBP-C protein levels" Where are those "normal levels" documented in the paper?

The discussion has been changed.

Response to Reviewer 3

We would like to thank the reviewer for his/her comments and critiques on our. We have performed new experiments to address your points.

Please note that all changes in the manuscript are marked with yellow.

Referee #3 (Comments on Novelty/Model System):

AAV9 mediated delivery of AONs in a knockin mouse model is already optimal and would be the choice for human therapy of this cardiac disease. The technical execution of the study and its model leaves a bit to be desired.

Referee #3 (General Remarks):

Gedicke-Hornung et al. Rescue of cardiomyopathy through U7snRNA-mediated exon skipping in Mybpc3 -targeted knock-in mice

Exon skipping mediated by antisense oligoribonucleotides (AON) is promising, but has not yet been evaluated for cardiac genetic diseases. Using a Mybpc3 targeted knock-in (KI) mouse model of hypertrophic cardiomyopathy (HCM), carry a homozygous G>A transition in exon 6, adeno-associated virus-mediated delivery of AONs was tested that evoke exon-skipping resulted in an expected Mybpc3 splice isoform (Var-4) and abolished cardiac dysfunction and prevented left ventricular hypertrophy in new-born mice but failed to affect young adult mice. This study represents the very first description of a molecular therapy aimed to "genotypically" correct a serious and medically unmet common human disorder.

Major

Fig. 1A. Indication of the size on the size marker is lacking.

Done.

Fig. 2A. Indication of the size on the size marker is lacking.

Done.

Fig. 2B, C. Loading controls (e.g. tubulin, gapdh or the like) for the Western blots are lacking.

Done.

Fig. 2D, E. Although the immunofluorescence localization studies in (neonatal?) mouse KI myocytes are appreciated, questions that arise are to what extent the likely developmental and technical differences between cultured myocytes and the myocardium in situ are to be expected.

Additionally, it was not clear to what extent the AAV transduction produces "supraphysiological" levels of Var-4, disrupting the "normal" pattern of Var-4. A more insightful analysis would include confocal analyses on intact myocardium WT, KI and AON-treated KI mice or adult myocytes.

It is known for long that the stochiometry of the sarcomere is very well respected, even after additional delivery of transgene (either after cell transfection or transduction or in transgenic mice). In other words, there is no evidence in the literature that gene transfer of a sarcomeric component is supra-overexpressed; in contrast, there is a body of evidence (many from the group of Jeff Robbins) that exogenous proteins partially or fully replace endogenous sarcomeric proteins in a way that the sarcomere stochiometry remains unchanged. The rest is degraded. Furthermore, Figures 2D, 2E show that not all cells are transduced, which is a good indication of no excessive expression.

We performed some IF of cardiac sections from KI mice treated with AAV9-U7-AON5+6. This shows that the sarcomere pattern is very well preserved (new Fig S6 of Supporting information).

We also evaluated the efficiency of heart transduction in vivo (new Fig S5B of Supporting information):

Fig. 4A. Fig. 4A, B. Indication of the size on the size marker is lacking. Loading controls for the Western blots are lacking.

We have now added the molecular size of the markers in Figure 4A. The loading control of Figure 4B was actually already in the Figure (total ERK).

Fig. 4B indicates that an AAV6 was used for a subset of experiments, but Fig. S3 depicts characteristics of an AAV serotype 9. Was either one a typo? If not, what differences in efficiency of exon skipping could exist between AAV6 and AAV9?

We are sorry that it was not very clear. There is no typo. Actually, and as explained in the text and in the methods, we used AAV serotype 6 for transduction of cultured cardiac myocytes and serotype 9 for a heart tropism in vivo. It has been shown before by others that AAV6 is superior in cultured cardiac myocytes, AAV9 superior in whole mice. Therefore both experiments are complementary.

Fig. 5A. Indication of the size on the size marker is lacking.

Done.

The most intriguing part of the study includes the phenotypic differences between adult vs new-born AON treated mice (Fig.5C and Fig.6B), where neonatal mice displayed benefit from exon-skipping but young adults failed to display phenotypic benefit despite more or less equally efficient exon-skipping. The conclusions - difference between neonatal and young adult mice - seem preliminary. The authors should first exclude that differences in escalating doses of AAV could reach a threshold of efficiency of exon skipping after which phenotypic benefit occurs, or, alternatively, waiting for more prolonged time periods could yield different results. One straightforward analysis that is required is whether the functional benefit recorded in 1 day-old mice (Fig. 6) is maintained throughout their lifespan or at least well into adulthood.

We thank the reviewer for his/her very important comment. We decided to evaluate the effect of treatment over a longer period (55 days). These data are now included in New Fig 9. Although we saw a complete rescue 7-15 days after administration of the AAV9-U7snRNA-AON5+6 *in vivo*, the rescue of the cardiac phenotype was not obvious anymore 55 days after treatment (new Fig 8). It should be noted, though, that the functional data in these young mice (to the best of our knowledge not systematically studied so far) are not easy to interpret. WT animals showed a marked reduction in FAS over time and this was the same, albeit at a lower level, in AAV9-injected KI mice. In contrast, FAS remained reduced in KI. From the similar pattern of WT and AAV-KI, the data can as well be interpreted as showing that the therapy was in fact effective even for the extended period. We have discussed this issue in the revised version.

2nd Editorial Decision 18 April 2013

Thank you for the submission of your revised manuscript to EMBO Molecular Medicine. We have now received the enclosed reports from the referees who were asked to re-assess it. Please accept my apologies for not getting back to you sooner.

As you will see, although referee #1 does not support publication mainly due to the uncertain clinical translation in humans, together with our chief editor and the second referee, we feel that the data is innovative and provides the 1st proof of principle that exon skipping may be a therapeutic option in cardiac genetic disease. As such, we are pleased to inform you that we will be able to accept your manuscript pending the following final amendments:

-Please modify the abstract and main text to make evident that the therapeutic effect observed is only transient.

***** Reviewer's comments *****

Referee #2 (Comments on Novelty/Model System):

Reasons are as in previous review

Referee #2 (General Remarks):

The new version of the manuscript includes new data in response to the reviewers' comments. The

new data however does not provide further demonstration of the therapeutic utility of the approach. On the contrary, the observation of the treated animals until day 55 indicate that the therapeutic effect is transient.
The claim of 'rescue of cardiomyopathy" in the paper title is therefore an overstatement.
The failure to detect variant 4 in human cardiac tissue is also worrying, and calls for a demonstration that it can be produced by exon skipping in cultured human cardiomyocytes.
Statements in the abstract and the discussion that no efficient AON mediated exon skipping has been obtained in the heart before this work, overlooks the work by M Wood's group where very significant levels of skipping are demonstrated, Yin et al 2001 and Betts et al 2012

Referee #3 (General Remarks):

no further remarks
