# Supplementary material for: Rescue of cardiomyopathy through U7snRNA-mediated exon skipping in Mybpc3-targeted knock-in mice
Source: EMBO Mol Med. 2013 May 29;5(7):1060–77. doi: 10.1002/emmm.201202168 (PMC3721478; doi:10.1002/emmm.201202168)
Supplement: Supplementary file 2 [file emmm0005-1060-SD2.pdf]

Supporting Information

Gedicke-Hornung, Behrens-Gawlik et al.

- **Figure S1.** Evidence for an alternative *Mybpc3* variant mRNA in wild-type mouse cardiac myocytes.
- **Figure S2.** Evaluation of Var-4 *Mybpc3* mRNA during mouse development.
- **Figure S3.** Effect of 2OMePS-modified AONs on *Mybpc3* mRNAs and cMyBP-C proteins in WT and KI cardiac myocytes.
- **Figure S4.** Evaluation of the *Mybpc3* mRNA species in wild-type cardiac myocytes.
- **Figure S5.** Tissue distribution of GFP after systemic administration of AAV9 in a *Mybpc3*-targeted knock-in mouse.
- **Figure S6.** Immunofluorescence analysis of cardiac sections after systemic administration of AAV9 in *Mybpc3*-targeted knock-in mice.
- **Figure S7.** Variant-4 rescues the phenotype of *Mybpc3*-targeted KI mice in the heart *in vivo*.
- **Figure S8.** Evaluation of AAV9 particles in ventricular tissue of KI injected mice.

Figure S1

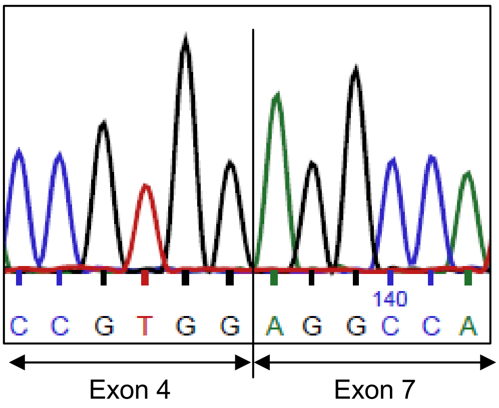

Figure S1. Evidence for an alternative *Mybpc3* variant mRNA in wild-type mouse cardiac myocytes.
RNA was extracted from neonatal wild-type mouse cardiac myocytes. After reverse transcription, two rounds of PCR were performed with primers complementary to exons 4 and 7 of *Mybpc3*.

The sequencing confirmed the skipping of exons 5 and 6, and therefore the presence of an alternative spliced *Mybpc3* mRNA isoform in cardiac myocytes from WT mice.

Figure S2

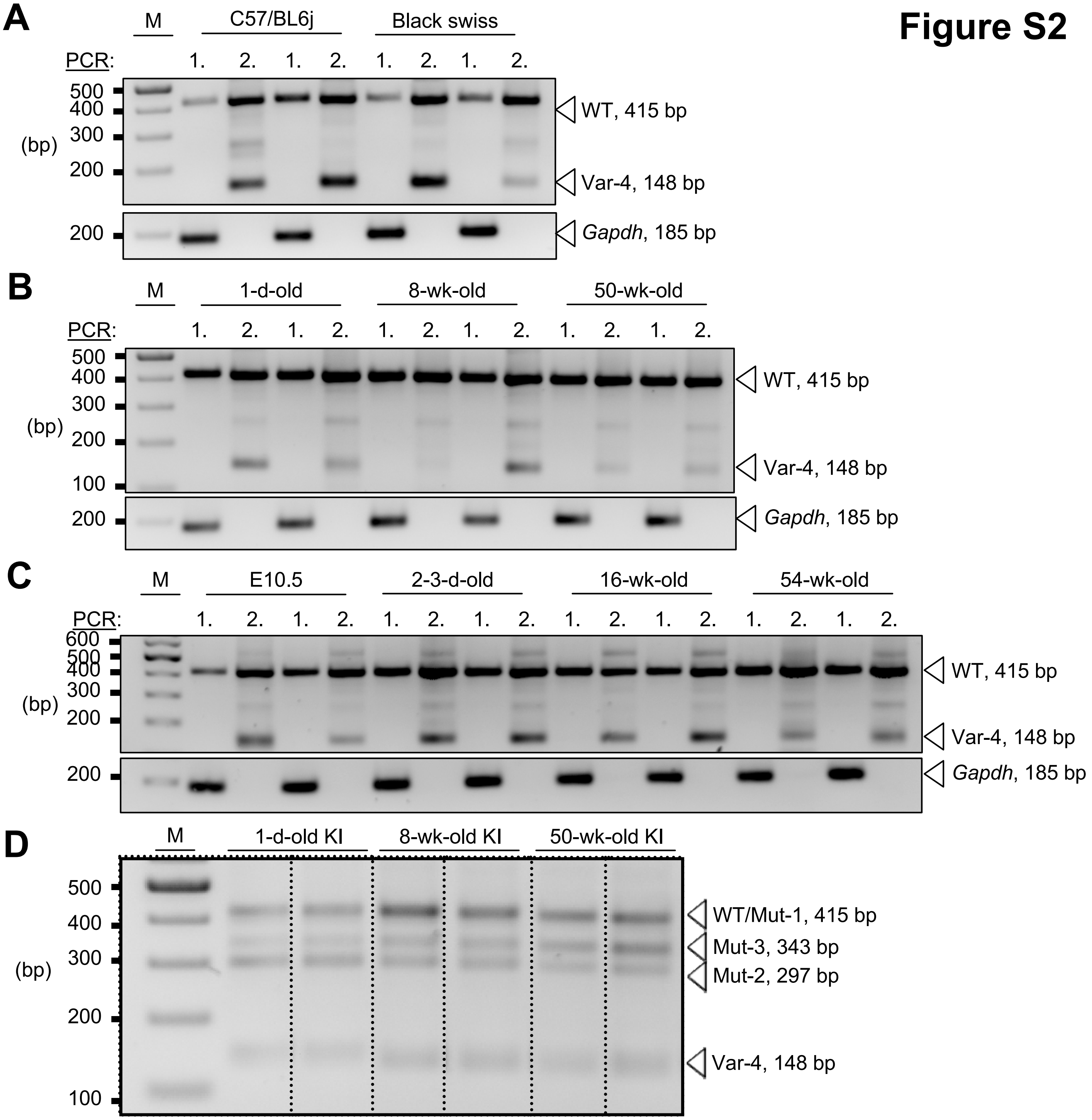

Figure S2. Evaluation of Var-4 *Mybpc3* mRNA during mouse development.

A) RT-PCR from RNA extracted from cardiac myocytes isolated from neonatal C57BL/6J or Black swiss wild-type mice after the first (1.) and second (2.) round of PCR using primers located in exons 4 and 9 of *Mybpc3* and in *Gapdh*.

B) RT-PCR from RNA extracted from ventricular tissue of wild-type Black swiss mice at different postnatal development after the first (1.) and second (2.) round of PCR using primers located in exons 4 and 9 of *Mybpc3* or in *Gapdh*.

C) RT-PCR from RNA extracted from cardiac tissue isolated from wild-type C57BL/6J mice at different times of embryonic and postnatal development after the first (1.) and second (2.) round of PCR using primers located in exons 4 and 9 of *Mybpc3* and in *Gapdh*.

D) RT-PCR (1 round of PCR only) from RNA extracted from cardiac tissue of *Mybpc3*-targted knock-in (KI) mice at different postnatal windows using primers located in *MYBPC3* exons 4 and 9.

The expected fragments are indicated by arrowheads. Abbreviation: M, 100-bp molecular weight marker.

These data showed that Var-4 is detected at a low level during the entire development in both C57BL/6J and Black swiss wild-type mice. These data support the view that it is a natural splicing variant of *Mybpc3*.
Furthermore, Var-4 mRNA was also detected in young and old KI mice with no major different intensity.

Figure S3

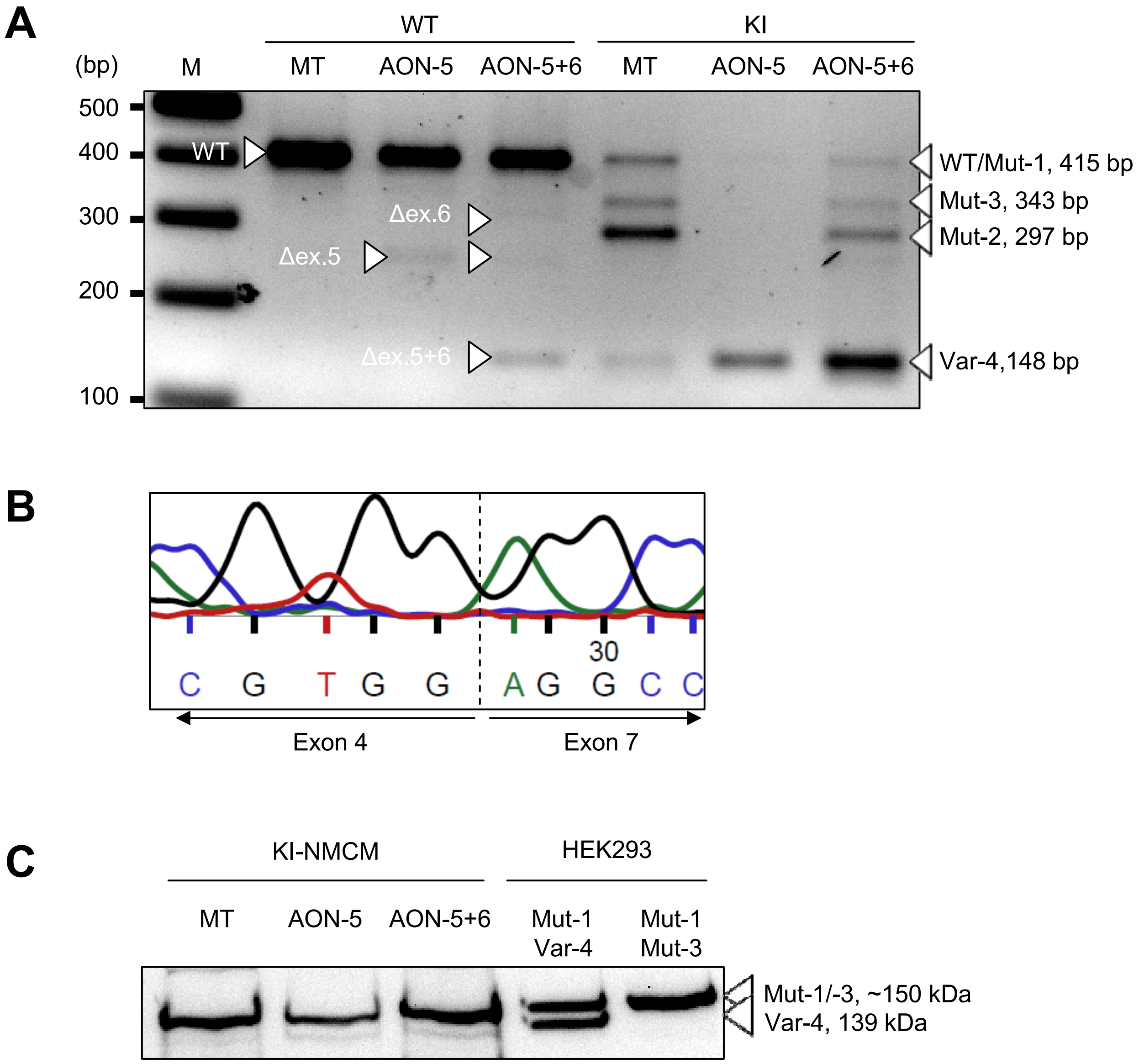

Figure S3. Effect of 2OMePS-modified AONs on *Mybpc3* mRNAs and cMyBP-C proteins in WT and KI cardiac myocytes.

Cardiac myocytes were isolated from wild-type (WT) and knock-in (KI) neonatal mice and either mock-transfected (MT) or transfected with antisense oligoribonucleotides directed against exon 5 (AON-5) or against exon 5 and exon 6 (AON-5+6) for 8 days. AONs were modified with 2'-O-methyl phosphorothioate (2OMePS).

A) RT-PCR performed with primers located in exons 4 and 9 of *Mybpc3*. The expected PCR fragments are indicated by arrowheads.

B) Validation of Var-4 in KI mice by sequencing analysis.

C) Representative Western blot of proteins derived from HEK293 cells and KI cardiac myocytes (KI NMCM). HEK293 cells were transiently transfected for 24 h with plasmids encoding Mut-1, Mut-3 and Var-4. KI NMCMs were either mock-transfected (MT) or treated for 8 days with AON-5 or AON-5+6. Western blot was stained with an antibody directed against the N-terminus of cMyBP-C. The expected protein fragments are indicated by arrowheads. Abbreviation: M, 100-bp molecular weight marker.

Figure S4

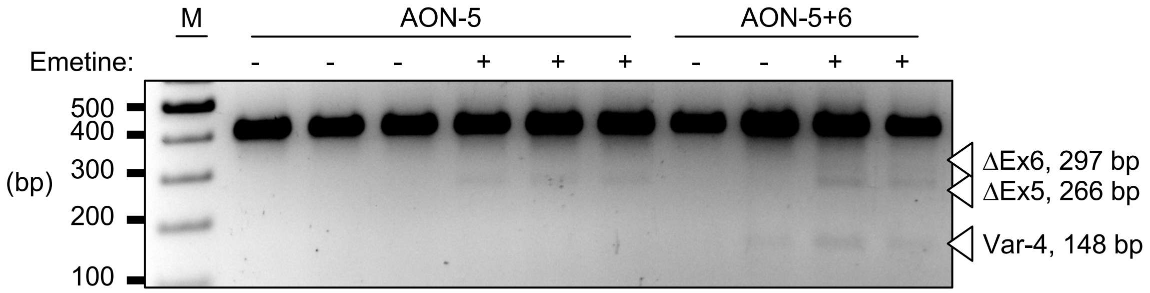

Figure S4. Evaluation of the *Mybpc3* mRNA species in wild-type cardiac myocytes.
Cardiac myocytes were isolated from wild-type neonatal mice and transduced for 72 h with adeno-associated virus serotype 6 (AAV6-U7snRNA-AON-5 or AAV6-U7snRNA-AON-5+6). At the end of the experiment, cells were treated (+) or not (-) with 300 µg/ml of the translation inhibitor emetine to prevent degradation of the nonsense mRNAs. The nonsense *Mybpc3* mRNAs deleted of either exon 5 (ΔEx5) or exon 6 (ΔEx6) were stabilized after emetine treatment, suggesting that they are normally degraded by the nonsense-mediated mRNA decay. On the other hand, Var-4 mRNA deleted of both exons 5+6 was stable and detected in baseline condition 72 h after transduction with AAV6-U7-AON-5+6 in a wild-type sample. The expected amplicons are indicated by the arrowheads. Abbreviation: M, 100-bp molecular weight marker.

These data showed that the exon skipping strategy mediated by AAV-based gene transfer of AONs works in wild-type neonatal mouse cardiac myocytes, and that the skipping of the single exon 5 or the single exon 6 results in nonsense mRNAs, which are normally degraded by the nonsense-mediated mRNA decay (NMD). When the NMD is blocked by the translation inhibitor emetine, the nonsense mRNAs were detected.

Figure S5

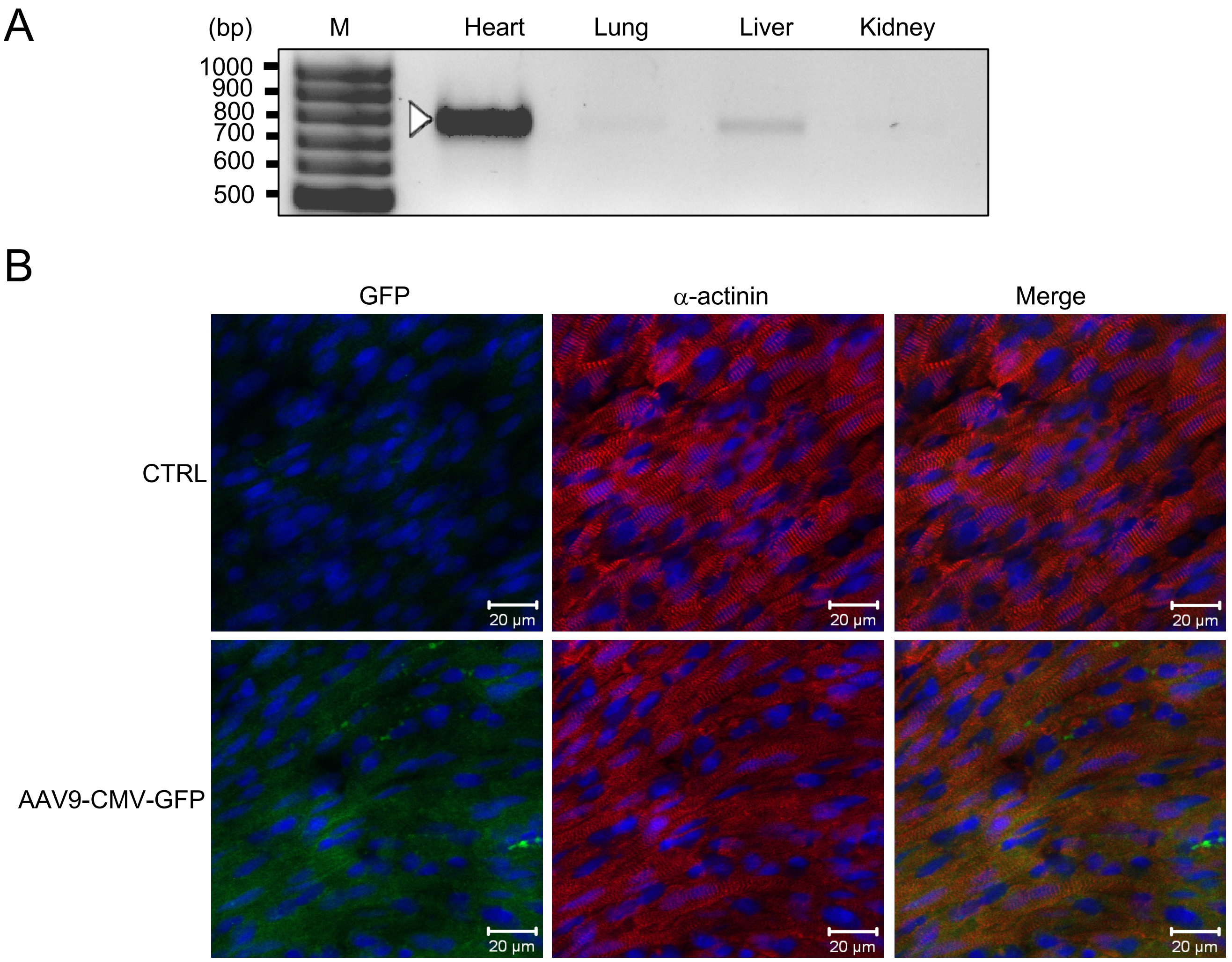

Figure S5. Tissue distribution of GFP after systemic administration of AAV9 in a *Mybpc3*-targeted knock-in mouse.
A) A 4-wk-old KI mouse received adeno-associated virus (AAV) serotype 9 encoding GFP ($7.6x10^{10}$ vg) by systemic administration into the tail vein. RT-PCR analysis was performed on RNA extracted from different tissues (heart, lung, liver and kidney) 4 weeks after AAV injection. For PCR amplification primers located in the GFP sequence were used. The expected amplicon is indicated by the arrowhead. Abbreviation: M, 100-bp molecular weight marker.
B) 1-day-old KI mouse received AAV9-CMV-GFP ($2x10^{11}$ vg) by systemic administration into the temporal vein for 7 days. 8-day-old control mouse (CTRL) did not receive anything. Hearts were extracted, rinsed in high K+, embedded in tissue Tek, and 10-µm-thick sections were performed. Sections were stained with primary antibodies directed against GFP (Santa Cruz, 1:200) and α-actinin (SIGMA, 1:800) and with the secondary antibodies anti-rabbit Alexa Fluor-488 (1:600) and anti-mouse Alexa Fluor-546 (1:600). Nuclei were stained with DRAQ5^{TM} (1:800). Slides were embedded in Mowiol and immunofluorescence analysis was performed by confocal microscopy using a Zeiss Axiovert microscope with a 40x-oil objective. Confocal images were recorded with a Zeiss LSM 710 system.

These data showed that i) GFP is more expressed in the heart than in the other organs, supporting the cardiotropism of the AAV serotype 9; ii) transduction efficiency of AAV9-CMV-GFP at a dose of $2x10^{11}$ gave a complete transduction of the heart 7 days after systemic administration.

Figure S6

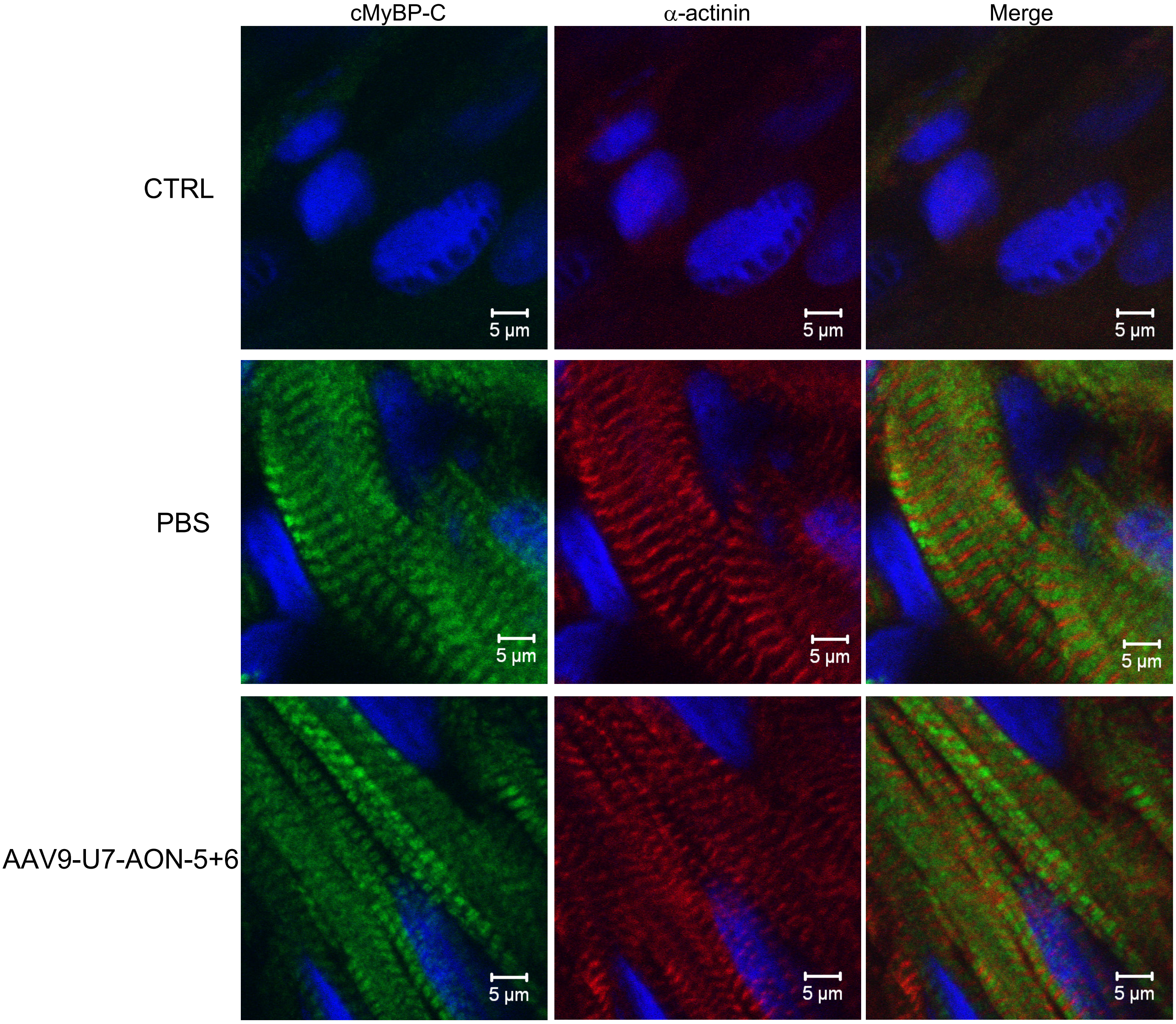

Figure S6. Immunofluorescence analysis of cardiac sections after systemic administration of AAV9 in *Mybpc3*-targeted knock-in mice.

1-day-old KI mice received either AAV9-U7-AON-5+6 ($2x10^{11}$ vg) or PBS by systemic administration into the temporal vein for 7 days. Hearts were extracted, rinsed in high K+ and embedded in tissue Tek, and 10-µm-thick sections were performed. Sections were stained with primary antibodies directed against the MyBP-C motif of cardiac myosin-binding protein C (cMyBP-C, 1:200) and α-actinin (1:800, Sigma) and with the secondary antibodies anti-rabbit Alexa Fluor-488 (1:600, Molecular Probes) and anti-mouse Alexa Fluor-546 (1:600, Molecular Probes). Nuclei were stained with DRAQ5TM (1:800, Molecular Probes). Control (CTRL) was stained only with the secondary antibodies. Slides were embedded in Mowiol and immunofluorescence analysis was performed by confocal microscopy using a Zeiss Axiovert microscope with a 100x-oil objective. Confocal images were recorded with a Zeiss LSM 710 system.

These data showed that cMyBP-C is organized in doublets in the A-band of the sarcomere and in alternation with α-actinin located at the Z-disk of the sarcomere. No major difference in the sarcomeric pattern was observed between the PBS-and AAV9-U7-AON-5+6 injected mice. This suggests that the stochiometry of the sarcomere is well preserved.

Figure S7

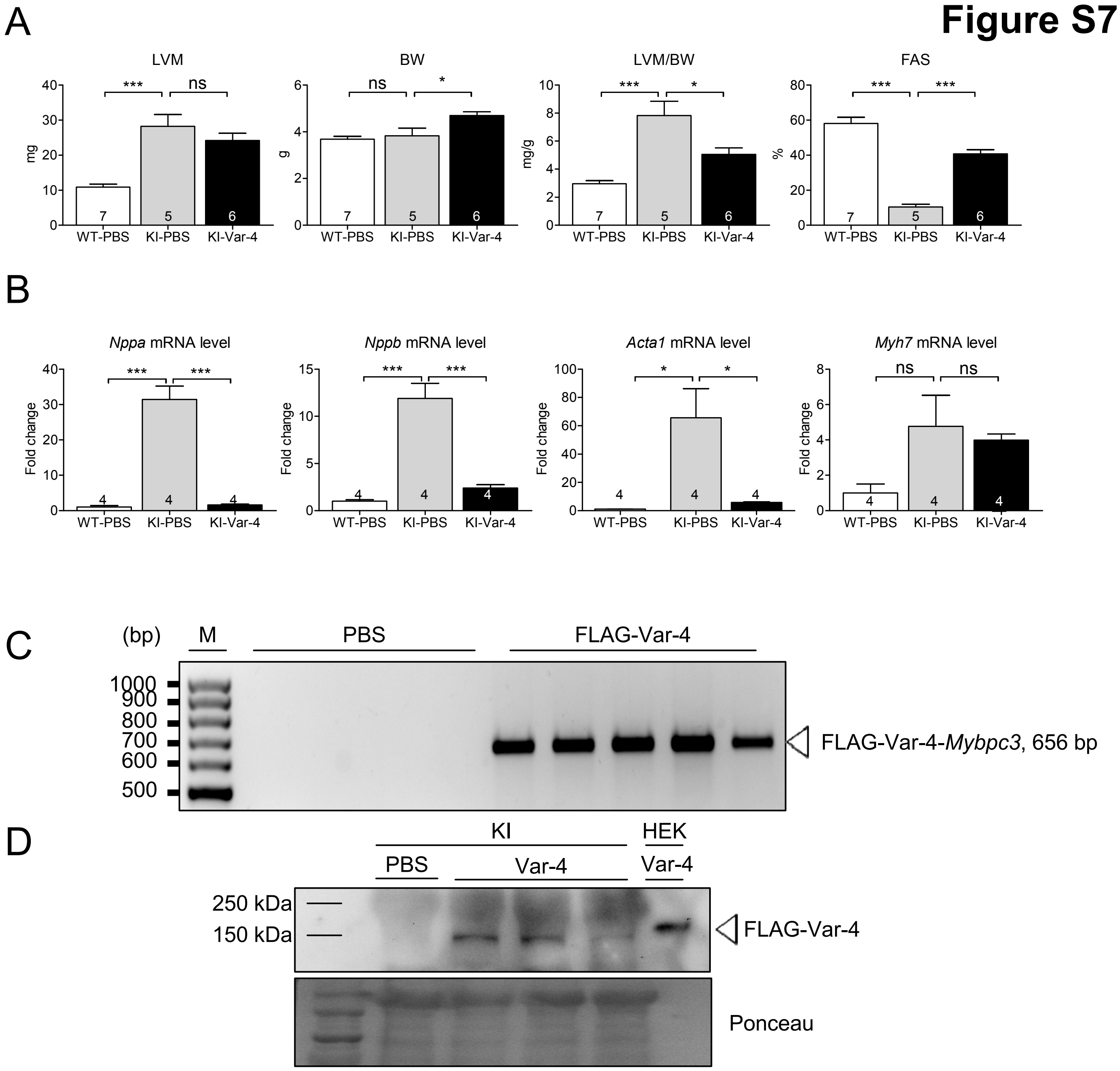

Figure S7. Variant-4 rescues the phenotype of *Mybpc3*-targeted KI mice in the heart *in vivo*.
1-day-old *Mybpc3*-targeted KI and WT mice received PBS or adeno-associated virus serotype 9 (AAV9) encoding FLAG-Var-4 under the control of the CMV promoter ($2x10^{11}$ vg) by systemic administration into the temporal vein. Echocardiographic analyses and molecular analyses were performed 7 days post-injection.
A) Left ventricular mass (LVM), body weight (BW), LVM/BW ratio and and fractional area shortening (FAS).
B) RT-qPCR analyses for atrial natriuretic peptide (*Nppa*), brain natriuretic peptide (*Nppb*), and α-skeletal actin (*Acta1*) normalized to GαS.
C) RT-PCR analysis of FLAG-Var-4 performed with primers located in the FLAG sequence and *Mybpc3* exon 9.
D) Western blot of ventricular tissue extract of AAV9-FLAG-Var-4- and PBS-treated KI mice stained with the anti-FLAG antibody. Var-4 was detected at 139 kDa. Positive control corresponds to protein extract of HEK293 cells transfected with FLAG-Var-4 cDNA plasmid. The Ponceau is shown below.
Values are expressed as mean±SEM. **P*<0.05 and ****P*<0.001 vs KI-PBS, one-way ANOVA and Bonferroni post-hoc test. Number of animals is indicated in the bars. M indicates 100-bp molecular weight marker.

These data showed that the expression of Var-4 prevents LVH and accumulation of *Acta1* on the one hand, and on the other hand restores cardiac function and normal levels of *Nppa* and *Nppb* mRNA in the KI hearts. These data suggest that Var-4 is not toxic *in vivo* and supports the exon skipping approach using AON-5+6 *in vivo* to rescue the phenotype of KI mice.

Figure S8

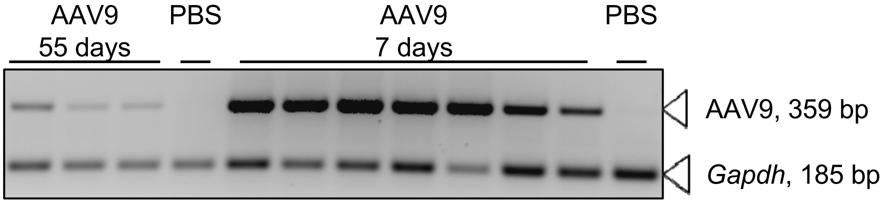

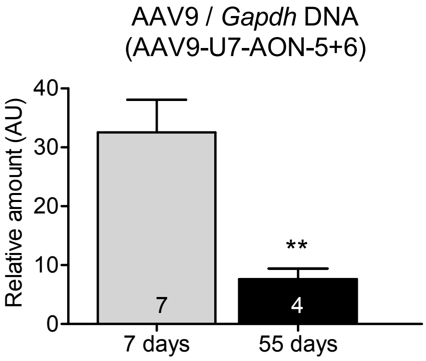

Figure S8. Evaluation of AAV9 particles in ventricular tissue of KI injected mice.
Neonatal KI mice received $2x10^{11}$ vg of adeno-associated virus (AAV9-U7-AON5+6) via the temporal vein, which corresponds to a mean dose of $1.44x10^{14}$ vg/kg of body weight. Ventricular tissues were removed 55 days or 7 days after AAV9 administration, and genomic DNA was extracted using Trizol or the Extract-N-AmpTM Tissue PCR kit (SIGMA) according to manufacturer's instructions. Multiplex PCR was performed on 20 ng DNA using primer pairs for AAV9 and for *Gapdh* (for AAV9: 5'-AGT GGC CAA CTC CAT CAC TA-3' and 5'-CAC AGA TGC TCA GAC CAC TTT TGC GGA AGT-3', and for *Gapdh*: 5'-ATT CAA CGG CAC AGT CAA G-3' and 5'-TGG CTC CAC CCT TCA AGT-3'). On the left panel is shown the results of the PCR on 1% agarose gel and on the right panel the quantification of the AAV9 DNA particles normalized to *Gapdh* DNA. **P<0.01 vs 7 days, Student's t-test.

These data showed that AAV9 particles were detected in all mice that received AAV9 and were 4-fold lower 55 days than 7 days after AAV9 administration. No AAV9 particles were detected in PBS-treated mice.
